# PD-1/PD-L1 axis induced host immunosuppression via PI3K/Akt/mTOR signalling pathway in piglets infected by *Glaesserella Parasuis*

**DOI:** 10.1186/s12917-024-03993-1

**Published:** 2024-04-06

**Authors:** Jingyang Li, Siyu Liu, Qiaoli Dong, Yunjian Fu, Yamin Sun, Ronghui Luo, Xinyue Tian, Ling Guo, Wei Liu, Yinsheng Qiu, Qirong Lu, Chun Ye, Bingbing Zong, Shulin Fu

**Affiliations:** 1 Hubei Key Laboratory of Animal Nutrition and Feed Science, Wuhan Polytechnic University, Wuhan, 430023 PR China; 2 Hubei Collaborative Innovation Center for Animal Nutrition and Feed Safety, Wuhan, 430023 PR China

**Keywords:** *Glaesserella parasuis*, Piglets, Spleen, Immunosuppression, Proteomics

## Abstract

*Glaesserella parasuis*, an important respiratory bacterial pathogen, causes Glässer’s disease in piglets, with potential immunosuppression. We established a piglet infection model and explored the immunosuppression mechanism to improve our understanding of the host immune response to *G. parasuis*. Twenty piglets were randomly divided into two groups (*n* = 10). The infection group was intraperitoneally challenged with 2 × 10^8^ CFU of *G. parasuis* in 2 mL TSB. The control group was intraperitoneally injected with equivalent TSB. After 72 h, the piglets were sacrificed, and spleen tissue was collected. PD-1/PD-L1 expression was determined. The splenocytes were isolated to detect CD3^+^ T, CD3^+^CD4^+^ T, CD3^+^CD8^+^ T and CD3^−^CD21^+^cell differentiation. Via data-independent acquisition (DIA), we compared the proteomics of healthy and infected spleen tissues. *Glaesserella parasuis* modified CD3^+^ T, CD3^+^CD4^+^ T, CD3^+^CD8^+^ T and CD3^−^CD21^+^ cell differentiation and PD-1/PD-L1 expression in the spleen. The infection group had 596 proteins with significant differences in expression, of which 301 were significantly upregulated and 295 downregulated. Differentially expressed proteins (DEPs) were mainly related to immune responses. This is the first study on PD-1/PD-L1 expression in the spleen associated with immunosuppression in a piglet model to explore the protein changes related to immune responses via DIA.

**Supplementary Information:**

The online version contains supplementary material available at 10.1186/s12917-024-03993-1.

## Background

*Glaesserella parasuis* (*G. parasuis*) is one of the most important respiratory pathogens in pigs and colonises the upper respiratory tract [1]. Infection of pigs with *G. parasuis* can cause Glässer’s disease, a severe systemic disease, resulting in large economic losses [2] caused by polyserositis, arthritis and meningitis [3]. So far, the infection mechanism of *G. parasuis* remains unclear. Some virulence-related factors have been reported. The capsular polysaccharides of *G. parasuis* is important virulence factors, providing the ability of avoiding recognition and killing by the host immune system [4]. Upregulation of occludin by cytolethal distending toxins enhances *G. parasuis* adhesion to the respiratory tract cells [5]. The *htrA* gene is associated with the survival and pathogenicity of *G. parasuis* [6], and the pathogen´s outer membrane protein P2 induces proinflammatory cytokine production in porcine alveolar macrophages [7]. In a previous study, the deletion of the two-component system QseBC weakened the virulence of *G. parasuis* in a murine acute infection model and adhesion to host cells [8]. Deletion of the *crp* gene effectively attenuates the virulence of *G. parasuis* [9]. Thus, studies on the pathogenic and immune mechanisms of *G. parasuis* are crucial to develop control strategies for *G. parasuis*.

Infection with *G. parasuis* can cause changes in protein expression in the spleen tissue, leading to dysfunctions in the host’s immune response. Proteomics is an important method for studying changes in protein expression after host infection with pathogenic bacteria [10], exploring the structures and functions of proteins [11]. Proteomics can reveal the mechanism of disease occurrence [12] and can be applied in drug development and design, screening novel drug targets [13]. The spleen is an important immune organ and crucial for innate immunity to occur [14]. In this study, we used proteomics to determine whether *G. parasuis* modulates host innate inflammatory cell responses in the spleen.

Host resistance to macrophage phagocytosis of *G. parasuis* infection requires the coordinated efforts of innate and adaptive immune cells [15]. Multiple cell types of the adaptive immune system respond to *G. parasuis* infection [16]. However, the T cells might be the principal antigen-specific cells responsible for the containment of *G. parasuis* infection [17]. Whilst CD3, CD4 and CD8 cells are the surface markers of T cells [18], the B cells are theatypical memory B cells, verified by the low expression of CD21 [19]. In a previous study, B cells with the CD21phenotype were observed in various bacterial infections [20] and viral infections [21]. Thus, in this study, T and B cell differentiation was explored to determine the adaptive immune responses after infection of piglets with *G. parasuis*.

We investigated the protein changes in the spleens of piglets infected with *G. parasuis* by proteomics methods and verified the expression of some key proteins and signalling pathways. Our findings will help address the molecular mechanisms of immunosuppression induced by *G. parasuis* and provides some molecular targets to control *G. parasuis* infection.

## Results

### *G. parasuis* modified the blood biochemical parameters and routine blood test, triggered inflammatory cytokine secretion and induced spleen tissue damage

After the piglets were challenged by *G. parasuis*, the blood biochemical parameters were determined at 24, 48 and 72 h. The levels of T-Bil, AST and D-Bil were significantly upregulated, whereas those of ALB, TC, GLU, HDL-C, LDL-C, γ-GT and CK were downregulated in the infection group compared to the control group (*p* < 0.05) (Table 1).

**Table 1:** Detection of the blood biochemical parameters

Item	Control	24 h GPS	48 h GPS	72 h GPS	SEM	* P* value		
	(A)	(B)	(C)	(D)		B vs. A	C vs. B	D vs. B
T-Bil(µmol/L)	3.01	3.85	10.10	3.74	1.01	< 0.001	< 0.001	< 0.001
TP(g/L)	46.80	43.90	45.17	46.01	0.48	0.041	0.207	0.526
ALB(g/L)	27.24	24.08	22.20	20.78	0.75	0.001	< 0.001	< 0.001
AST(U/L)	65.00	121.00	115.00	192.00	14.05	0.001	0.001	< 0.001
ALT(U/L)	41.00	36.00	23.00	19.00	2.95	0.279	0.002	0.001
ALP(U/L)	577.00	373.00	296.00	266.00	46.93	0.068	0.020	0.013
TC(mmol/L)	1.81	1.21	1.12	1.09	0.11	0.032	0.018	0.015
TG(mmol/L)	0.32	0.32	0.25	0.39	0.02	0.868	0.005	0.007
GLU(mmol/L)	4.50	2.60	2.10	0.47	0.44	< 0.001	< 0.001	< 0.001
Ca(mmol/L)	2.23	2.03	2.04	2.31	0.05	0.096	0.110	0.479
IP(mmol/L)	2.85	3.00	2.87	2.76	0.04	0.110	0.817	0.313
CRE(µmol/L)	92.72	90.26	76.06	52.77	4.81	0.155	< 0.001	< 0.001
HDL-C(mmol/L)	0.56	0.13	0.21	0.18	0.05	< 0.001	< 0.001	< 0.001
LDL-C(mmol/L)	0.98	0.47	0.55	0.50	0.08	0.011	0.023	0.014
UA(µmol/L)	2.67	4.58	10.29	5.84	1.72	0.129	< 0.001	0.023
γ-GT(U/L)	68.00	56.00	43.00	35.00	3.92	0.004	< 0.001	< 0.001
CK(U/L)	912.00	692.00	690.00	515.00	45.09	0.002	0.002	< 0.001
D-Bil(µmol/L)	0.47	1.99	5.57	2.42	0.57	0.001	< 0.001	< 0.001
LDH(U/L)	1067.90	1067.00	1359.20	1501.90	76.39	0.994	0.042	0.007

*GPS* *G. parasuis*

In the routine blood test, the WBC were significantly decreased in the infection group compared to the control group at 24 h (*p* < 0.001) (Table 2). The RBC, NE and LYM levels were significantly decreased, and MONO were significantly increased in the infection group compared to the control group from 24 to 72 h (*p* < 0.05) (Table 2).

**Table 2:** Detection of the routine blood test

Item	Control	24 h GPS	48 h GPS	72 h GPS	SEM	* P* value		
	(A)	(B)	(C)	(D)		B vs. A	C vs. A	D vs. A
WBC(10^9^/L)	19.36	8.47	14.82	16.39	1.27	<0.001	0.011	0.064
RBC(10^9^/L)	6.51	5.59	4.58	3.50	0.35	0.01	<0.001	<0.001
HGB(g/L)	121.00	134.00	101.00	75.00	7.87	0.363	0.186	0.011
PLT(10^9^/L)	349.00	189.00	156.00	114.00	28.33	0.001	0.315	0.038
NE(10^9^/L)	5.69	3.32	3.49	3.03	0.37	0.005	0.008	0.003
LYM(10^9^/L)	10.84	2.53	4.94	3.21	1.01	<0.001	<0.001	<0.001
MONO(10^9^/L)	1.08	3.29	4.50	5.50	0.53	0.008	0.001	<0.001
EOS(10^9^/L)	0.24	0.29	0.82	0.98	0.11	0.759	0.003	0.001

*GPS* *G. parasuis*

**Fig. 1:**
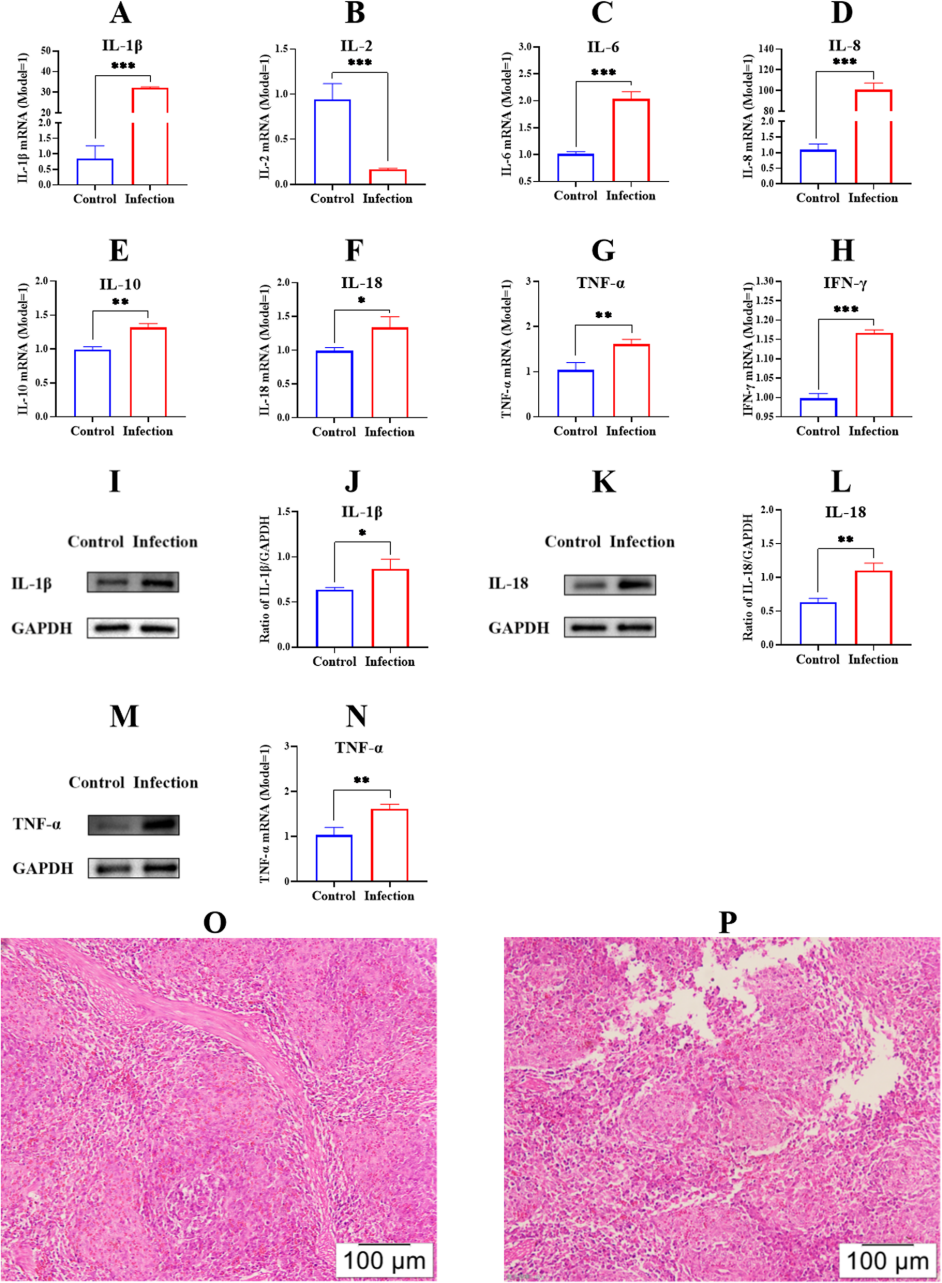
Detection of the cytokines expression and histopathological analysis of the piglet spleens in the control group and the infection group. The RNAs from PMNPs were extracted and reverse-transcribed into cDNA. The production of cytokines of IL-1β (Fig. 1A), IL-2 (Fig. 1B), IL-6 (Fig. 1C), IL-8 (Fig. 1D), IL-10 (Fig. 1E), IL-18 (Fig. 1F), TNF-α (Fig. 1G), and IFN-γ (Fig. 1H) at mRNA levels were determined by RT-PCR. Total proteins of spleen tissue were extracted and IL-1β (Fig. 1I and J), IL-18 (Fig. 1K and L), and TNF-α (Fig. 1M and N) expression at the protein level were measured by Western blot. O: the histopathological analysis of control group; P: the histopathological analysis of infection group of. ^*^ *p* < 0.05; ^**^ *p* < 0.01^*^; ^***^ *p* < 0.001

The inflammatory cytokine expression levels in the spleen were determined by RT-PCR and the Western blot method. Based on the results, the mRNA levels as well as IL-1β, IL-6, IL-8, IL-10, IL-18, TNF-α and IFN-γ were significantly upregulated, and the IL-2 level was decreased in the infection group compared to the control group (*p* < 0.05) (Fig. 1A-H). We also determined inflammation cytokine expression at the protein level. The pathogen promoted IL-1β, IL-18 and TNF-α expression at the protein level compared to the control group (*p* < 0.05) (Fig. 1I-N), suggesting an induction of the inflammation immune responses in the piglets.

When the piglets were infected with *G. parasuis*, severe tissue damage was observed in the spleen (Fig. 1P), whereas the control group did not show any pathological damage (Fig. 1O). In the infection group, the spleen displayed inflammatory responses, with haemorrhage and necrosis. The number of splenic white pulp lymphocytes was decreased, the red pulp cord became narrow, and the splenic sinus was congested (Fig. 1P).

#### *G. parasuis* triggered PD-1/PD-L1 expression in the spleens of piglets

**Fig. 2:**
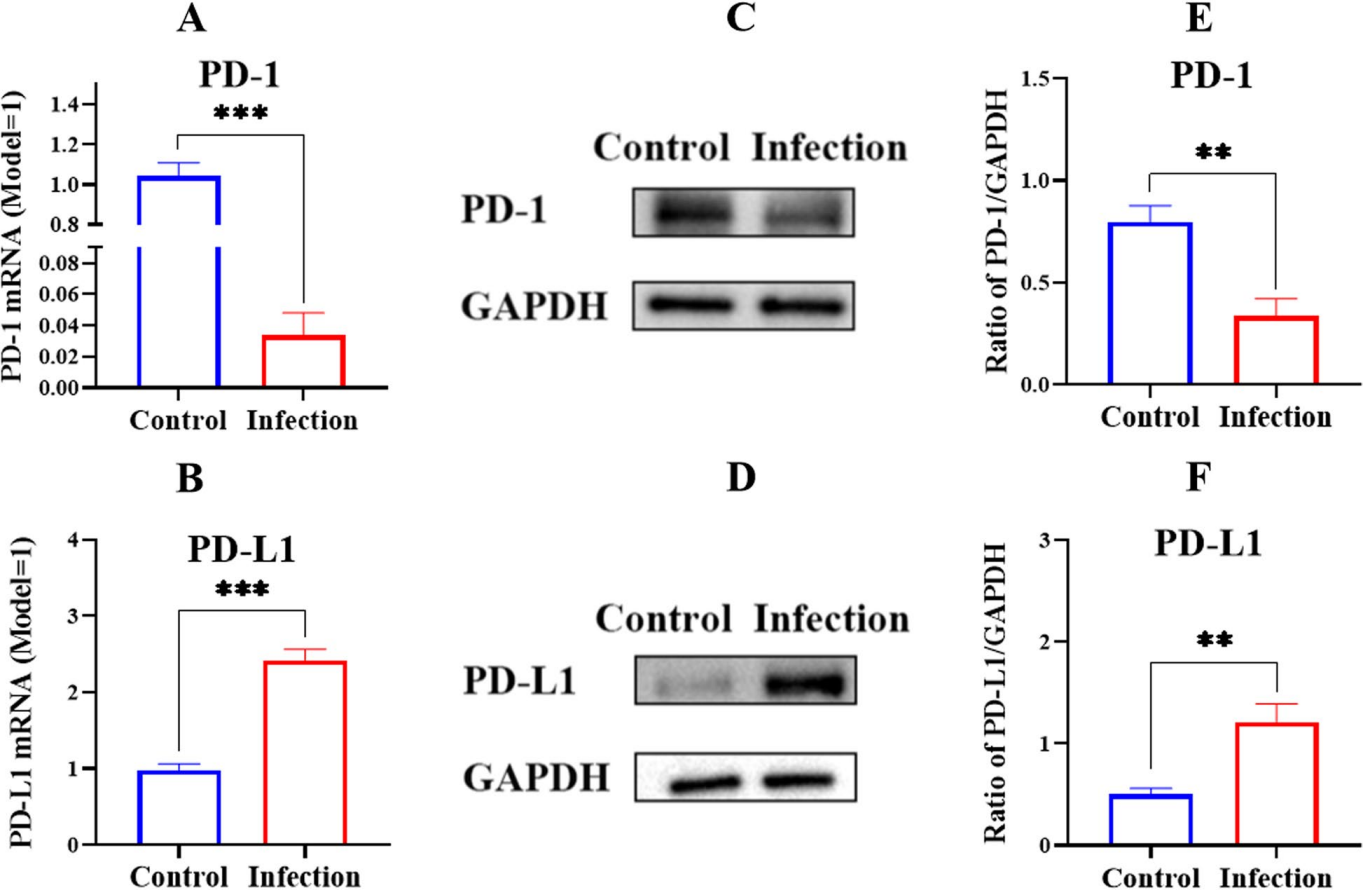
Determination of the PD-1/PD-L1 expression at mRNA and protein levels by RT-PCR and western blot respectively. **A** PD-1 expression at mRNA level; **B** PD-L1 expression at mRNA level; **C**, **E** PD-1 expression at protein level; **D**, **F** PD-L1 expression at protein level. ^**^ *p* < 0.01^*^; ^***^ *p* < 0.001

After the piglets were infected with *G. parasuis*, PD-1/PD-L1 expression at mRNA and protein levels was determined by RT-PCR and Western blot, respectively. The PD-L1 expression at the mRNA level was significantly increased, and the PD-1 expression level was decreased in the spleens from infected animals compared to those from the control group (*p* < 0.001) (Fig. 2A and B). Furthermore, *G. parasuis* induced PD-L1 protein expression and inhibited PD-1 protein expression in the spleens of animals from the infection group compared to those from the control group (*p* < 0.01) (Fig. 2C and F).

#### *G. parasuis* attenuated CD3^+^ T, CD3^+^CD4^+^ T, CD3^+^CD8^+^ T and CD3^−^CD21^+^ cell proportions in the spleen

**Fig. 3:**
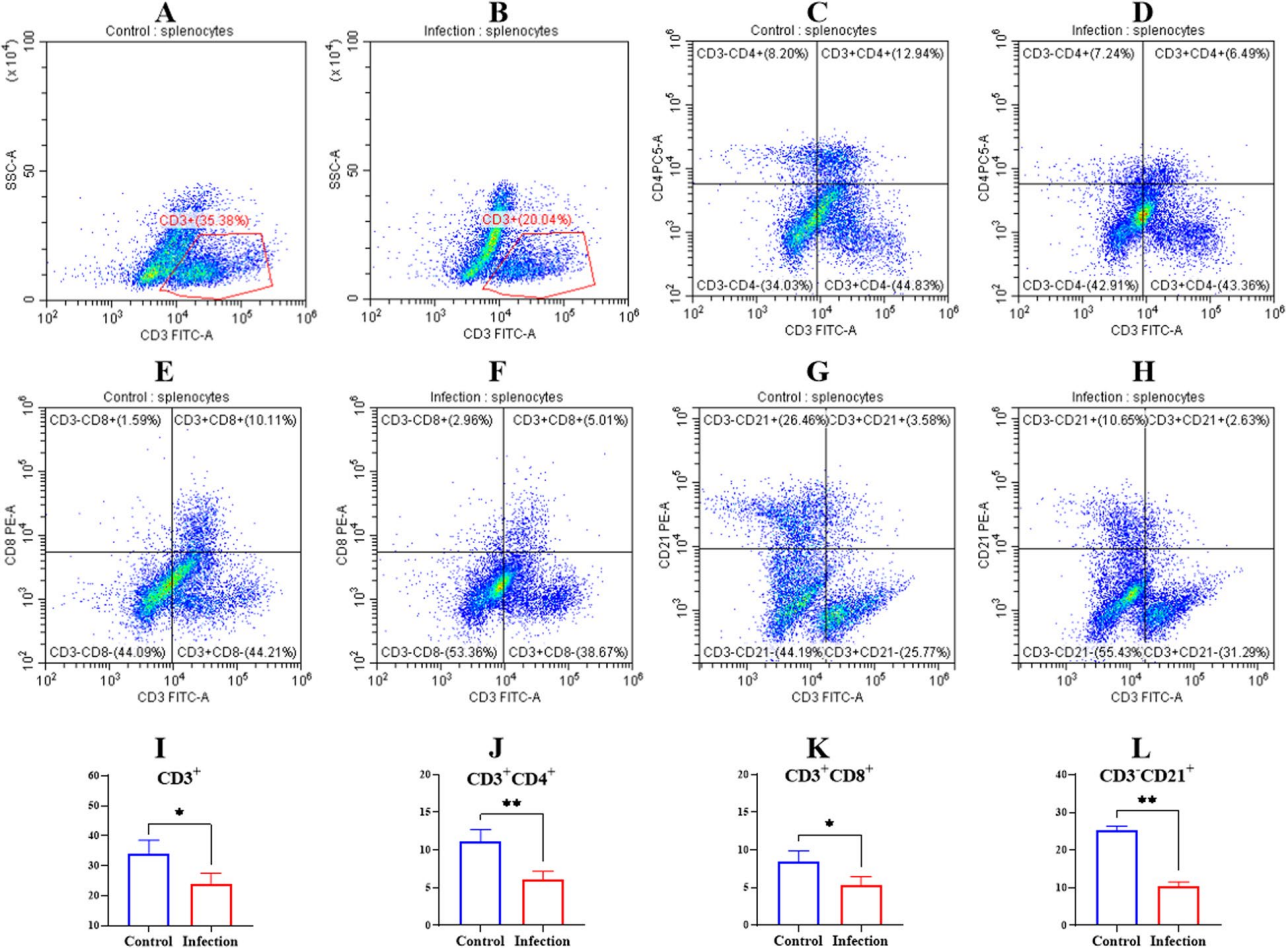
Determination of the CD3^+^ T cells, CD3^+^CD4^+^ T cells, CD3^+^CD8^+^ T cells, and CD3^−^CD21^+^ cells proportion in spleen by flow cytometry. The splenocytes were isolated and the splenocytes differentiation was determined by flow cytometry. A, C, E, G: CD3^+^ T cells, CD3^+^CD4^+^ T cells, CD3^+^CD8^+^ T cells, CD3^−^CD21^+^ cells in the control group respectively; B, D, F, H: CD3^+^ T cells, CD3^+^CD4^+^ T cells, CD3^+^CD8^+^ T cells, CD3^−^CD21^+^ cells in the infection group respectively. ^*^ *p* < 0.05; ^**^ *p* < 0.01^*^

The T and B cell immune responses play important roles in regulating host adaptive immunity. After the piglets were challenged with *G. parasuis*, the CD3^+^ T, CD3^+^CD4^+^ T, CD3^+^CD8^+^ T and CD3^−^CD21^+^ cell proportions in the spleen were determined by flow cytometry. The CD3^+^ T cell proportion in the spleen in the infection group was significantly lower compared to that of the control group(*p* < 0.05) (Fig. 3B and I). Also, *G. parasuis* could reduce the CD3^+^CD4^+^ T, CD3^+^CD8^+^ T and CD3^−^ CD21^+^ cell proportions in the spleen compared to the control group (*p* < 0.01) (CD3^+^CD8^+^ T cells, *p* < 0.05) (Fig. 3D, F, H, J, K and L).

**Fig. 4:**
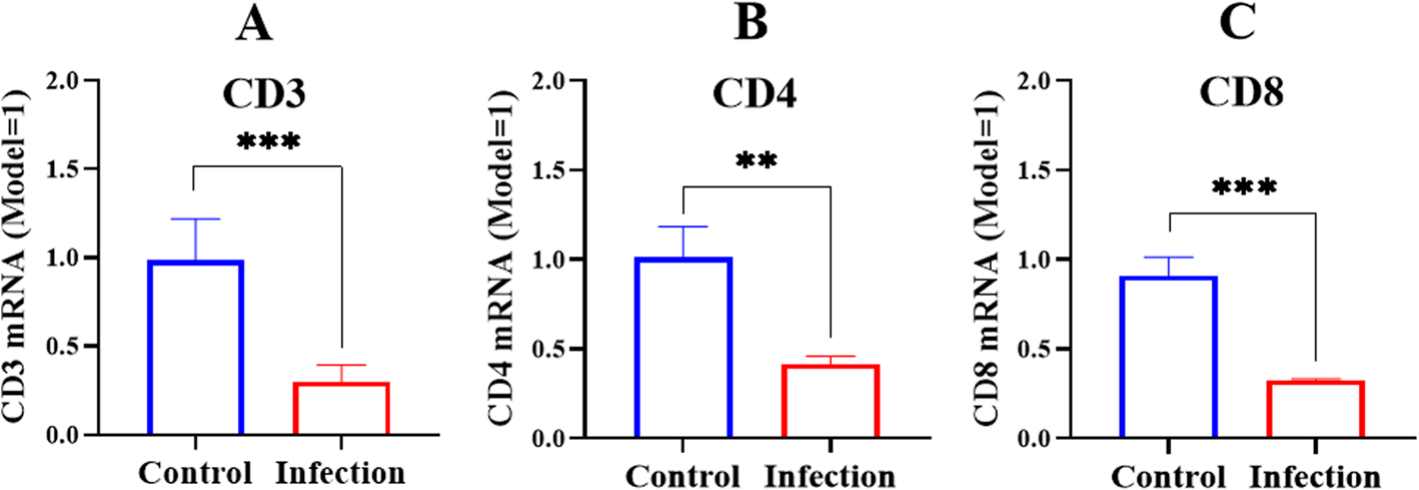
Determination of the CD3 (**A**), CD4 (**B**), CD8 (**C**) expression at mRNA level by RT-PCR. ^**^ *p* < 0.01^*^; ^***^ *p* < 0.001

We also determined the CD3, CD4, and CD8 expression at mRNA level. The results showed that *G. parasuis* reduced the CD3, CD4, and CD8 expression at mRNA level in the infection group compared to the control group (*p* < 0.001) (CD4, *p* < 0.01) (Fig. 4A, B and C).

#### *G. parasuis* infection contributes to protein dysregulation in spleen tissue

**Fig. 5:**
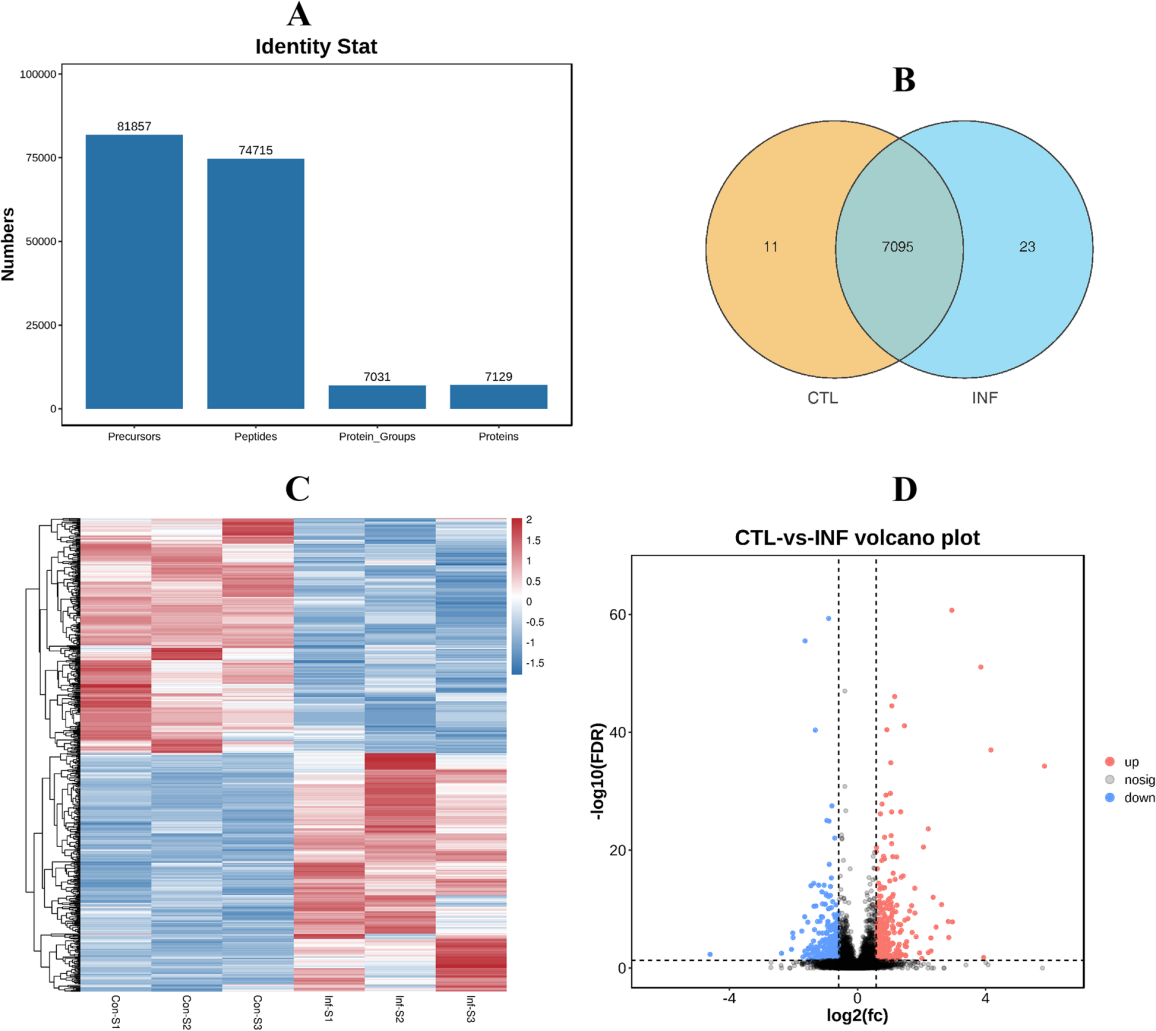
Dysregulated proteins of piglet spleens after *Glaesserella parasuis* infection. **A** Identified and quantified proteins from quantitative proteomics. **B** Venn diagram of protein groups verified from the infection and the control groups. **C** Heat map of dysregulated proteins from the infection and the control groups. **D** Volcano plot of dysregulated proteins. CTL (Con-S1, Con-S2, Con-S3): the control group; INF (Inf-S1, Inf-S2, Inf-S3):the infection group

To reveal the molecular mechanism of spleen inflammation induced by *G. parasuis*, the proteins from the spleens in the control and treatment groups were analysed using proteomics sequencing. The results showed that 74,715 peptides were verified, corresponding to 7,031 protein groups (Fig. 5A). Further, 7,091 protein groups were quantified in the infection group and the control group (Fig. 5B). Using |fold change|>1.5 and *Q* value < 0.05 as filtering criteria, 596 DEPs were obtained from the spleens of infected piglets, of which 301 DEPs were upregulated and 295 downregulated (Fig. 5C, D).

**Fig. 6:**
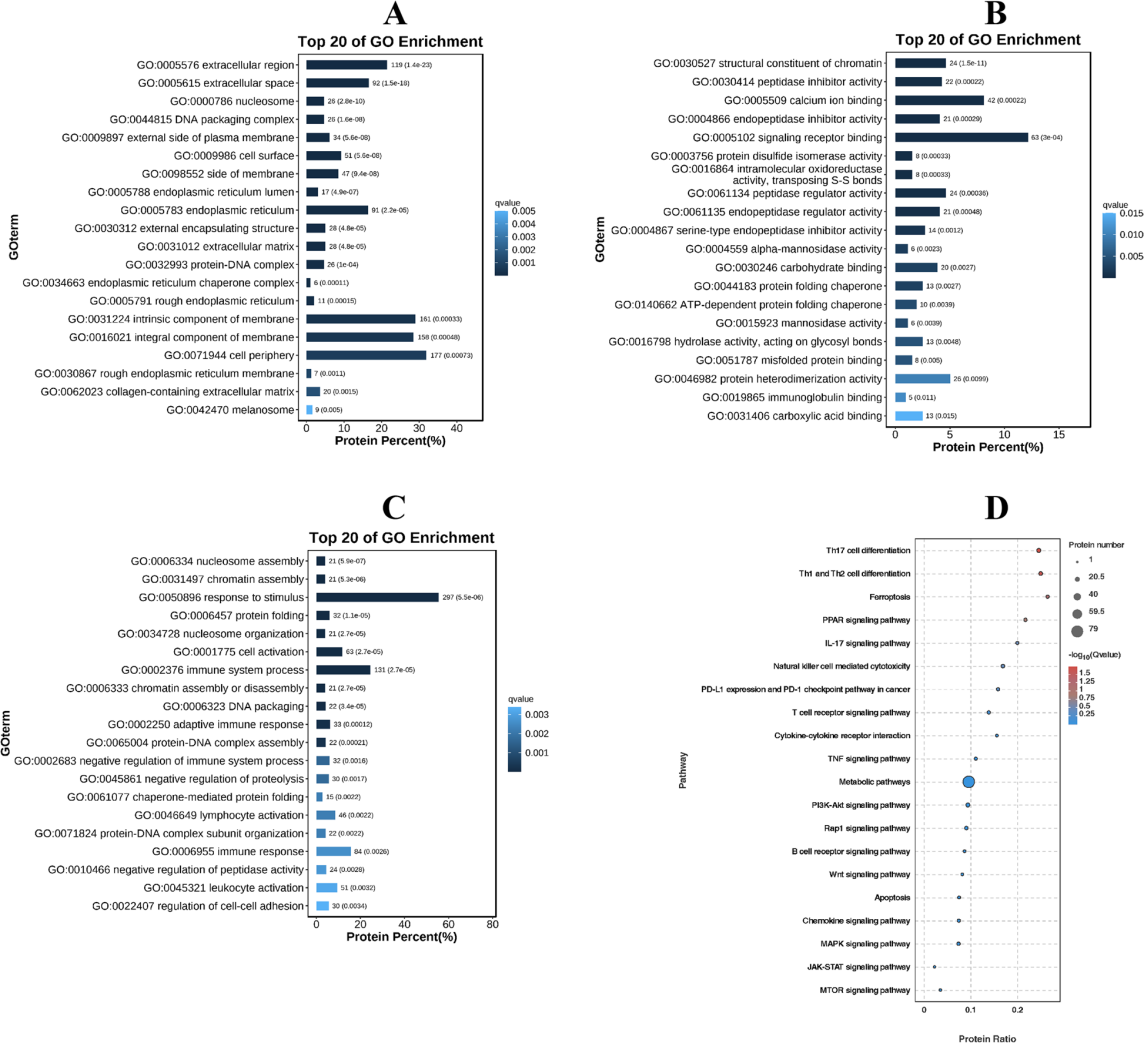
Go and KEGG analysis. GO functional enrichment analysis of the DEPs of the different categories (**A** cellular components; **B** molecular functions; **C** biological processes). **D** KEGG analysis of the dysregulated DEPs from the proteomics of the spleen

The GO enrichment analysis included three categories, namely cellular components, molecular functions and biological processes. In the first category, the DEPs were mainly involved in cell periphery and integral membrane components (Fig. 6A). Signalling receptor binding and response to stimulus were the dominant biological processes in which the DEPs were involved (Fig. 6B, C). The KEGG enrichment analysis showed that the dominant signalling pathways were metabolic pathways, Th17 cell differentiation, Th1 and Th2 cell differentiation, PI3K-Akt signalling pathway and mTOR signalling pathway (Fig. 6D).

#### *G. parasuis* induced PI3K/Akt/mTOR signalling pathway activation in piglet spleens

**Fig. 7:**
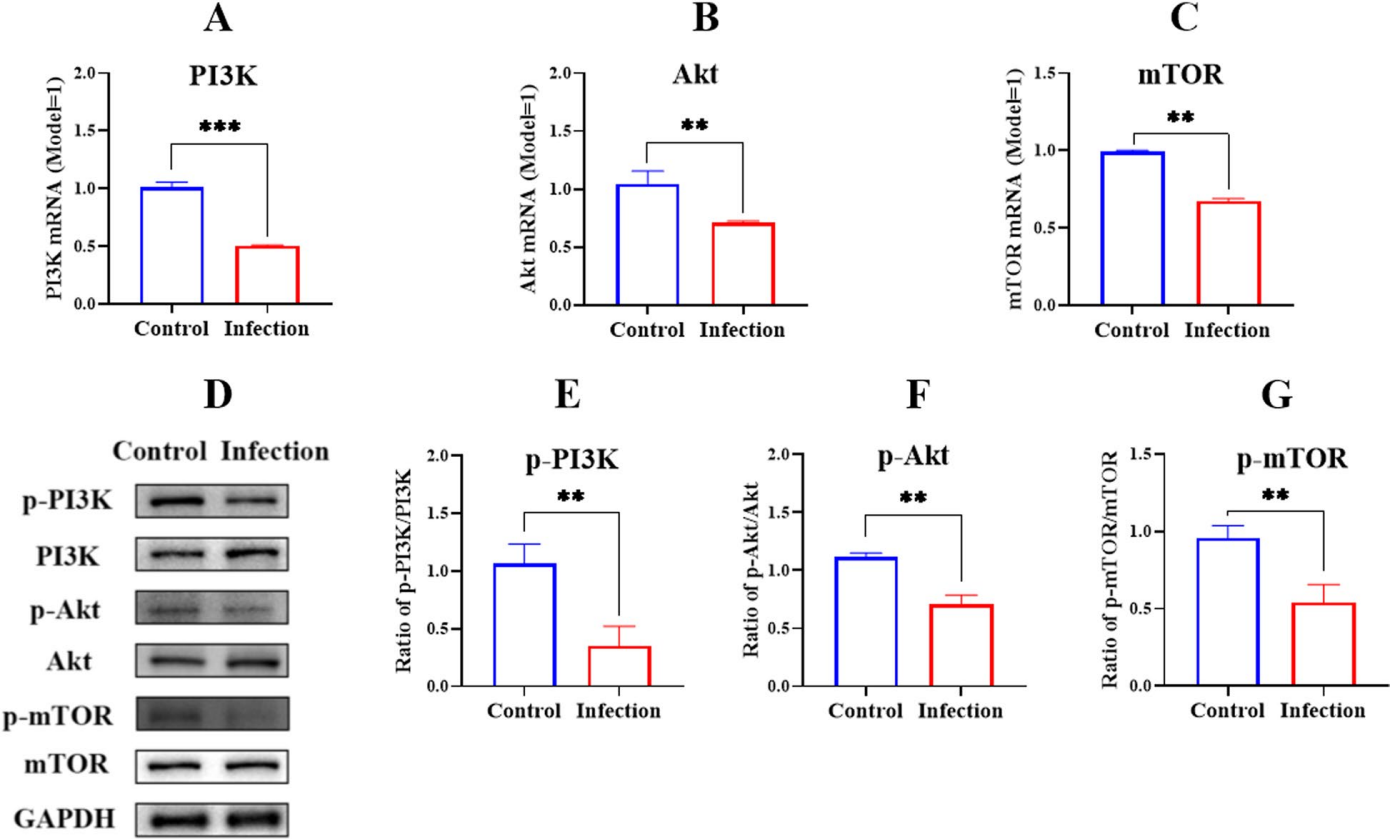
Determination of the PI3K/Akt/mTOR signaling pathway activation in the spleen by G. parasuis. The PI3K, Akt, and mTOR expressions at mRNA **A**-**C** and protein levels **D**-**G** were detected by RT-PCR and Western blot, respectively. **p < 0.01*; ***p < 0.001

We determined whether the PI3K/Akt/mTOR signalling pathway, which was also enriched in the KEGG pathway, was activated as a consequence of *G. parasuis* infection. Based on the results, the mRNA level as well as PI3K, Akt and mTOR expressions were decreased in the infection group compared to the control group (*p* < 0.01) (Fig. 7A, B and C). At the protein level, Infection with *G. parasuis* resulted in reduced p-PI3K, p-Akt and p-mTOR levels in the spleen compared to the control group (*p* < 0.01) (Fig. 7D, E, F and G).

## Discussion

The major functions of the spleen include the production of opsonised platelets and white blood cells, along with the removal of pathogenic microorganisms and antigens [22]. The spleen, as the largest immune organ and immunecentre [23], is prone to damage as a result of Glässer’s disease, aggravating the damage of other organs and increasing mortality. However, the mechanism of spleen injury caused by *G. parasuis* is still unclear. In this study, we applied proteomic methods to explore the spleen immune responses of piglets infected with *G. parasuis*.

Infection of the host with pathogenic bacteria can induce inflammatory responses and produces cytokines [24], and massive amounts of inflammatory cytokines could result in immune disorders and diseases [25]. The IL-1β is associated with the immune reconstitution inflammatory syndrome of chronic disseminated candidiasis [26], and TNF-α drives inflammatory responses by triggering cell death, instigating inflammatory immune reactions and disease development [27]. In another study, IL-18 controlled skin inflammation in the progression of Buruli ulcers caused by *Mycobacterium ulcerans* [28]. In the present study, we investigated cytokine production in the spleen induced by *G. parasuis*. Based on our findings, *G. parasuis* induced IL-1β, IL-18 and TNF-α secretion, which might be associated with spleen inflammation responses or inflammation damage.

Programmed cell death ligand 1 (PD-L1) plays important roles in maintaining immune homeostasis through inhibiting T cell immune responses via binding to programmed cell death protein 1 (PD-1) on T cells [29]. The PD-L1 leads to T-cell dysfunction or exhaustion, diminishing the intensity of antigen-specific T-cell responses in tumour tissues [30]. The upregulation of the expression of PD-L1 in the epithelial-mesenchymal transformation could trigger immunosuppression and escape, promoting cancer metastasis [31]. Although *G. parasuis* can induce host immunosuppression, the underlying mechanism is still unclear. We speculated that PD-1/PD-L1 plays important roles in inducing host immunosuppression by *G. parasuis*. Thus, we investigated PD-1/PD-L1 expression in the spleen induced by *G. parasuis* and found that *G. parasuis* triggered PD-L1 upregulation and PD-1 downregulation. This leads us to infer that PD-1/PD-L1 activation caused by *G. parasuis* is an important factor leading to host immunosuppression.

The cytokines secreted at the site of infection could recruit innate immune cells, thereby transmitting antigenic signals to adaptive immune cells such as B and T cells, resulting in immunosuppression and immune dysfunction [32]. Both T and B cells are key immune cells and play important roles in resisting diseases and maintaining immune balance [33]. In this study, we explored T and B cell differentiation in the spleens of piglets infected with *G. parasuis*. The proportions of CD3^+^ T, CD3^+^CD4^+^ T, CD3^+^CD8^+^ T and CD3^−^CD21 + cells in the spleen were decreased induced by *G. parasuis*, suggesting that *G. parasuis* affected the differentiation of T and B cells. In a previous study, abnormal T cell differentiation altered T cell functions through decreasing the capacity of the immune system to kill infected cells [34], which might be an important factor of immune escape after *G. parasuis* infection. Abnormal B cell differentiation might lead to multi-organ damage [35] or immune dysregulation [36]. Severe bacterial infections might lead to the attenuation of B cell percentages [37], which is consisted with our results. Thus, in our future study, we will investigate in detail the relationship between abnormal T and B cell differentiation and host susceptibility caused by bacteria to immune cell phagocytosis.

Previous research showed that PD-1/PD-L1 signalling could trigger PI3K/Akt/mTOR signalling pathway activation [38]. In this study, KEGG analysis showed that the PI3K-Akt signalling pathway was the main pathway in which DEPs were enriched. In similar studies, the PI3K/Akt/mTOR pathway was related to acute kidney injury triggered by cisplatin [39] and acute lung injury induced by bleomycin [40]. Airway inflammation and airway remodelling in chronic asthma are regulated by the PI3K-Akt signalling pathway [41]. Further, LncRNA TUG1 promotes the idiopathic pulmonary fibrosis progression of interstitial lung disease via activating the PI3K/Akt/mTOR pathway [42]. The is also evidence that bisphenol A inducesthe development of systemic lupus erythematosus in MRL/lpr mice, atypical autoimmune disease, which is related to the up-regulation of the PI3K/AKT/mTOR signalling pathway [43]. Spleen injury is caused by splenic ROS affecting PI3K/AKT/mTOR pathway-mediated autophagy in severe acute pancreatitis [44]. The PI3K/AKT/mTOR pathway is related to immunosuppression in septic mice, providing a new target for the treatment of sepsis [45]. Based on its important roles in inflammation responses, we investigated whether the PI3K/Akt/mTOR signalling pathway was activated in the spleen of piglets infected with *G. parasuis*. Although the PI3K/Akt/mTOR signalling pathway was activated, whether PI3K/Akt/mTOR signalling pathway activation is involved in host immunosuppression still needs to be investigated.

Overall, our results indicate that *G. parasuis* triggered cytokine production and PD-1/PD-L1 activation, induced spleen CD3^+^ T, CD3^+^CD4^+^ T, CD3^+^CD8^+^ T and CD3^−^CD21^+^ cell abnormal differentiation and activated the PI3K/Akt/mTOR signalling pathway. These findings deepen our understanding of the mechanism of host immunosuppression induced by *G. parasuis* and facilitate the development of some novel targets to control *G. parasuis* infection.

## Materials and methods

### Ethics statement

Animal studies were approved by the Animal Care and Use Committee of Wuhan Polytechnic University, Hubei Province, China (WPU202308001). All experimental animals were euthanized intravenously with pelltobarbitalumnatricum at the dosing of 80 mg/kg body weight at the end of the experiment.

### Bacteria and culture conditions

The serovar 5 *G. parasuis* SH0165 strain was isolated from a commercially obtained pig lung that showedarthritis, fibrinous polyserositis, hemorrhagic pneumonia and meningitis [46]. The SH0165 strain was grown in tryptic soy broth (TSB) (Difco Laboratories, USA) or on tryptic soy agar (TSA) (Difco Laboratories, USA) and supplemented with 10 µg/mLof NAD (Sigma, USA) and 10% foetal bovine serum (Sijiqing, Hangzhou, China) at 37 °C.

### Experimental design

Twenty 21-day-oldnaturally farrowed early-weaned (NFEW) piglets (Duroc × Landrace × large white), with a body weight of 4–5 kg, were purchased from the Wuhan Fenglongxin Breeding Professional Cooperative (Wuhan, China). The piglets were randomly divided into two groups, namely the control and the infection group. The piglets from the infection group were intraperitoneally challenged with 2 × 10^8^ CFU of *G. parasuis* in 2 mL TSB, whereas the control group only received the equivalent TSB thtough intraperitoneally. The piglets of both groups were monitored for 3 days.

### Blood biochemical parameters and routine blood testing

At 24, 48 and 72 h after the challenge, blood was collected to determine the blood biochemical parameters and for routine blood testing [47]. The blood was collected from the anterior venae cavae and placed into tubes containing ethylenediaminetetraacetic acid (EDTA). The blood samples were centrifuged at 3,000 × g for 15 min under 4 °C to isolate the plasma, which was used to determine the blood biochemical parameters using commercial kits (Shanghai Kehua Bio-engineering Co., Ltd., Shanghai, China). We determined the levels of total bilirubin (T-Bil), total protein (TP), albumin (ALB), aspartate aminotransferase (AST), alanine aminotransferase (ALT), alkaline phosphatase (ALP), total cholesterol (TC), triglycerides (TG), glucose (GLU), calcium (Ca), inorganic phosphate (IP), creatinine (CRE), high-density lipoprotein cholesterol (HDL-C), low-density lipoprotein cholesterol (LDL-C), uric acid (UA), γ-glutamyl transpeptidase (γ-GT), creatine kinase (CK), direct bilirubin (D-Bil) and lactate dehydrogenase (LDH). Anticoagulant blood was prepared for routine blood testing. White blood cells (WBC), red blood cells (RBC), haemoglobin (HGB), platelets (PLT), neutrophils (NE), lymphocytes (LYM), monocytes (MONO) and eosinophils (EOS) were determined using an automatic blood analyser (Hitachi HITEC 7100, Japan).

### Determination of cytokine secretion by real-time quantitative PCR (RT-PCR) and Western blot

At 72 h after *G. parasuis* challenge, blood samples were collected from all 10 piglets in each group for cytokine determination. The production of IL-1β, IL-2, IL-6, IL-8, IL-10, IL-18, TNF-α and IFN-γ in the serum was measured by RT-PCR, and IL-1β, IL-18 and TNF-α were determined by Western blot analysis. Peripheral blood monocytes (PMNPs) were obtained as described in our previous study [48]. The RNA from the PMNPs was extracted by using TRISOL reagent (Invitrogen, USA) and reverse-transcribed into cDNA by using reverse transcriptase (TaKaRa, Dalian, China). The cDNA synthesis was carried out using the SYBR Green PCR Kit (TaKaRa, Dalian, China) according to the manufacturer’s instructions. The transcriptions of each sample were repeated at least three times, using GAPDH as internal control. The primers used in this study for RT-PCR measurement are listed in Table 3.

**Table 3:** Primer sequences for qRT-PCR analysis

Gene		Nucleotide Sequence (5’-3’)	Tm (℃)	Length (bp)		accession numbers
IL-1β	Forward	TCTGCATGAGCTTTGTGCAAG	59.73	155	NM_001302388.2	
	Reverse	ACAGGGCAGACTCGAATTCAAC	60.87			
IL-6	Forward	CTTCTGGTGATGGCTACTG	52.66	134	AF518322.1	
	Reverse	TTGCCGAGGATGTACTTAA	50			
IL-8	Forward	ACAGCAGTAACAACAACAAG	50.18	117	AB057440.1	
	Reverse	GACCAGCACAGGAATGAG	53.17			
IL-10	Forward	CGTGGAGGAGGTGAAGAGTG	55.4	178	NM_214041.1	
	Reverse	TTAGTAGAGTCGTCATCCTGGAAG	55.6			
IL-18	Forward	AGTAACCATATCTGTGCAGTGT	53.95	155	AF191088.1	
	Reverse	TCTTATCACCATGTCCAGGAAC	53.04			
TNF-α	Forward	CGCTCTTCTGCCTACTGCACTTC	60.68	164	JF831365.1	
	Reverse	CTGTCCCTCGGCTTTGACATT	57.77			
IFN-γ	Forward	GAGGTTCCTAAATGGTAGCTCTGG	57.08	164	S63967.1	
	Reverse	TCTGACTTCTCTTCCGCTTTCTT	55.55			
IL-2	Forward	AGCCATTGCTGCTGGATTT	55.05	107	FJ543109.1	
	Reverse	AGCCTGCTTGGGCATGTAA	57.34			
PD-1	Forward	GCGGAATGTCAAGGAAACC	54.31	150	NM_001097431.1	
	Reverse	CTGTACCCGTGGAGGAGGA	59.14			
PD-L1	Forward	AATGGCGAGGAAGACCTGAA	56.24	137	NM_001025221.1	
	Reverse	CAGCAGTAAACCCCTGCATCT	57.62			
CD3	Forward	GTTTGCTGATGGTGGTGTA	52.4	144	NM_214227.1	
	Reverse	TGGGCTCATAGTCTGGATT	52.45			
CD4	Forward	AGCCTCAGTTACCGAGTT	52.74	138	KU248481.1	
	Reverse	ATCCTCTTGTCTTCCACTTC	51.14			
CD8	Forward	GTTACATCTCTGGTTACAAGG	49.89	139	NM_001001907.1	
	Reverse	AAGAAGACGGACATGAAGTT	50.65			
PI3K	Forward	TTGCTACAATCAATCGCCAGGAGAC	59.32	147	XM_021086552.1	
	Reverse	CTTCCCGTTGTTGCCATCGTTTG	59.67			
Akt	Forward	GGACGGGCACATCAAGATCACTG	60.84	126	NM_001159776.1	
	Reverse	TAGTCGTTGTCCTCCAGCACCTC	61.16			
mTOR	Forward	AGTACCTCCAGGACACCATGAACC	60.88	108	XM_003127584.6	
	Reverse	CAGACCTCACAGCCACAGAAAGC	60.97			
GAPDH	Forward	GGCACAGTCAAGGCGGAGAAC	61.89	105	NM_001206359.1	
	Reverse	AGCACCAGCATCACCCCATTTG	60.99			

The protein expression levels were determined by the Western blot method [49]. Total proteins of spleen tissue were extracted using RIPA (radio-immunoprecipitation) lysis buffer, and the protein concentration was measured using the Enhanced BCA Protein Assay Kit (Beyotime, Shanghai, China); subsequently, the proteins were separated using SDS-PAGE and electrotransferred to PVDF membranes. Following blocking by 5% skim milk, the blots were incubated with primary antibody of IL-1β, IL-18, TNF-α, PD-1, PD-L1, PI3K, p-PI3K, Akt, p-Akt, mTOR, p-mTOR, or GAPDH under 4 °C for 12 h, respectively. After washing with TBST five times, the blots were incubated with corresponding HRP Goat Anti-Rabbit IgG (IL-1β, IL-18, PI3K, p-PI3K, Akt, GAPDH) (Abbkine, Wuhan, China), or HRP Goat Anti-Mouse IgG (H + L) (TNF-α, PD-1, PD-L1, p-Akt, mTOR, p-mTOR) (ABclonal, Wuhan, China) under 37 °C for 1 h and subsequently treated with the ECL Enhanced Kit (ABclonal, Wuhan, China). The protein expression levels of IL-1β, IL-18, TNF-α, PD-1, PD-L1, PI3K, p-PI3K, Akt, p-Akt, mTOR and p-mTOR were quantified using the FluorChem™ FC2 AIC system (Alpha Innotech, USA).

### Isolation of the splenocytes from the piglets

The splenocytes were isolated as previously described, with some minor modifications [50]. Briefly, the piglets from all 10 piglets in each group were sacrificed on the 3rd day after *G. parasuis* challenge. The spleens were harvested aseptically and processed through gentle disruption using a sterile stainless-steel sieve and a glass pestle. The splenocytes were suspended in RPMI incomplete medium (Gibco, USA) and centrifuged for 30 min at 200 × g. The erythrocytes were lysed using lysing buffer (Biosharp, China) for 15 min. After washing five times using Hank’s balanced salt solution (HBSS) (Gibco, USA), the splenocytes were resuspended in complete RPMI medium (Gibco, USA) for flow cytometry analysis.

### Flow cytometry

The splenocytes were isolated to analyse cell differentiation via CytoFLEX SRT flow cytometry (Beckman, Suzhou, China) and incubated with Mouse Anti-Porcine CD3ε-FITC (SouthernBiotech, Birmingham, USA), Mouse Anti-Porcine CD4-SPRD (SouthernBiotech, Birmingham, USA), Mouse Anti-Porcine CD8a-PE (SouthernBiotech, Birmingham, USA) and Mouse Anti-Porcine CD21-PE (SouthernBiotech, Birmingham, USA). At least three independent samples were used, and each sample was repeated three times, and the data were obtained using the CytExpert SRT software.

### Proteome sample preparation

Spleen tissue samples of piglets from the infection and the control groups were collected and immediately ground in liquid nitrogen. Subsequently, the spleen samples were transferred into lysis buffer (1% SDS, 8 M urea, 1 mg/Ml protease inhibitor cocktail), vortexed and lysed for 30 min on ice, followed by homogenisation for 3 min in ice using an ultrasonic homogeniser. The homogenate was centrifuged at 12,000 rpm for 30 min at 4 ℃, and the supernatant was collected.

The protein concentration of the supernatant was measured using the BCA protein assay. Briefly, 100 µg of protein was transferred into a new Eppendorf tube, and the final volume was adjusted to 100 µL with 8 M urea. Afteradding 2 µL of 0.5 M TCEP to the tube for incubation at 37 ℃ for 1 h, we added 4 µL of 1 M iodoacetamideand incubated the mixture for 40 min. Five volumes of -20 ℃ pre-chilled acetone were added to precipitate the proteins overnight at -20 ℃. After washing twice with 1 mL of pre-chilled 90% acetone aqueous solution, the precipitates were re-dissolved in 100 µL of 100 mM TEAB. Sequence-grade modified trypsin (Promega, Madison, WI) was added at aratio of 1:50 to digest the proteins at 37 ℃ overnight. Subsequently, the peptide mixture was desalted by C18 ZipTip, quantified using thePierce™ quantitative colorimetric peptide assay and lyophilised by SpeedVac. The resultant peptide mixture was labelledwith the iTRAQ-8 PlexIsobaric Mass Tag Labelling Kit (ThermoFisher Scientific, MA, USA) according to the manufacturer’s instructions. The labelled peptide samples were then pooled and lyophilised in a vacuum concentrator. The peptide mixture was re-dissolved in buffer A (20 mM ammonium formate in water, pH 10.0, adjusted with ammonium hydroxide) and then fractionated by high-pH separation on an Ultimate 3000 system (ThermoFisher scientific, MA, USA) connected to a reverse phase column (XBridge C18 column, 4.6 mm x 250 mm, 5 μm, Waters Corporation, MA, USA).

High-pH separation was performed using a linear gradient, ranging from 5% B to 45% B in 40 min (B: 20mM ammonium formate in 80% ACN, pH 10.0, adjusted with ammonium hydroxide). The column was re-equilibrated at the initial condition for 15 min, with a column flow rate of 1 mL/min and a column temperature of 30℃. The fraction was dried using a vacuum concentrator.

### Nano-HPLC-MS/MS analysis

The obtained peptides were re-dissolved in solvent A (0.1% formic acid in water) and determined using the on-line nanospray LC-MS/MS with Orbitrap Fusion™ Lumos™ Tribrid™ coupled to the EASY-nLC 1200 system (Thermo Fisher Scientific, MA, USA). We loaded 4 µL of the peptide on the trap column (Thermo Fisher Scientific Acclaim PepMap C18, 100 μm x 2 cm) and the analytical column (Acclaim PepMap C18, 75 μm x 15 cm) and separated it with a 90 min-gradient from 5 to 32% B (0.1% formic acid in ACN). The column flow rate was maintained below 600 nL/min, with a column temperature of 40 °C. Anelectrospray voltage of 2 kV versus the inlet of the mass spectrometer was employed.

The mass spectrometer was run at data-dependent acquisition mode and automatically switched between MS and MS/MS mode. The parameters were as follows: (1) MS: scan range (m/z) = 350–1550; resolution = 60,000; AGC target = 4e5; maximum injection time = 50ms; charge states = 2–6; dynamic exclusion = 30 s; (2) HCD-MS/MS: resolution = 30,000; isolation window = 1.2; AGC target = 7e4; maximum injection time = 120ms; collision energy = 38.

### Data analysis

The tandem mass spectra were processed using the PEAKS Studio version X+ (Bioinformatics Solutions Inc., Waterloo, Canada). The PEAKS DB was set up and assumed trypsin as the digestion enzyme, searched with a fragment ion mass tolerance of 0.02 Da and a parent ion tolerance of 10 ppm. Carbamidomethylation (C) and iTRAQ 8plex (K, N-term) were specified as the fixed modifications. Oxidation (M) and deamidation (NQ) were specified as the variable modifications. Peptides which passed the 1% Qvalue cutoff were used to calculate the major group quantities with the MaxLFQ method. The RAW data was submitted to the Open Archive for Miscellaneous Data (OMIX) (OMIX005726, https://ngdc.cncb.ac.cn/omix/preview/JB8k13R5).

Differentially expressed proteins (DEPs) were analysed using Student’s t test and the Benjamini and Hochberg (BH) procedure. Subsequently, the DEPs were filtered with the selection criteria of fold change > 1.5 and Qvalue < 0.05 and mapped to the GO terms in the gene ontology database (http://www.geneontology.org/). The calculated *p* value was subjected to FDR correction (FDR ≤ 0.05 as a threshold). The genes were mapped to the KEGG database, and the calculated *p*-value was checked by FDR correction, using FDR ≤ 0.05 as the threshold.

### Histopathological analysis

Spleen tissue was fixed in 10% neutral buffered formalin and embedded in paraffin. Following cutting and staining with haematoxylin and eosin using a standard protocol, 4-µm tissue sections were examined using a light microscope.

### Statistical analysis

The experimental data are expressed as mean ± SD. Differences were analysed using analysis of One Way ANOVA. A value of *P* < 0.05 indicated statistical significance.

### Supplementary Information

**Supplementary Material 1.**

## Data Availability

The RAW data was submitted to the Open Archive for Miscellaneous Data (OMIX) (OMIX005726, http://ngdc.cncb.ac.cn/omix/preview/JB8k13R5).
