# Supplementary material for: PD-1/PD-L1 axis induced host immunosuppression via PI3K/Akt/mTOR signalling pathway in piglets infected by Glaesserella Parasuis
Source: BMC Vet Res. 2024 Apr 6;20:141. doi: 10.1186/s12917-024-03993-1 (PMC10998357; doi:10.1186/s12917-024-03993-1)
Supplement: Supplementary file 1 — Supplementary Material 1. [file 12917_2024_3993_MOESM1_ESM.pdf]

Fig 1

Fig 1.I:

IL-1β

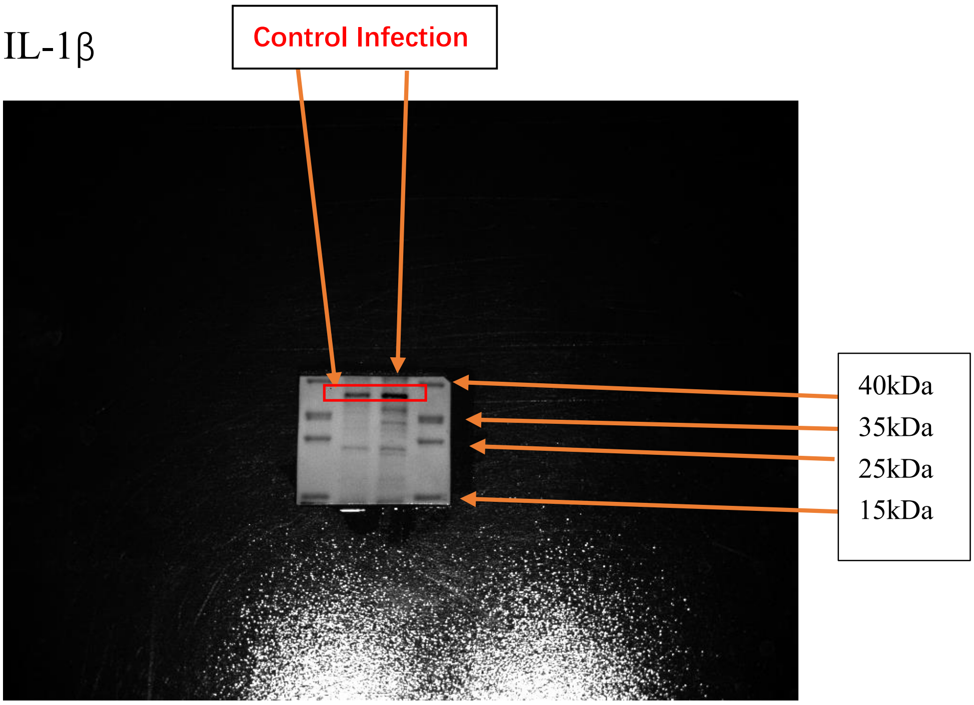

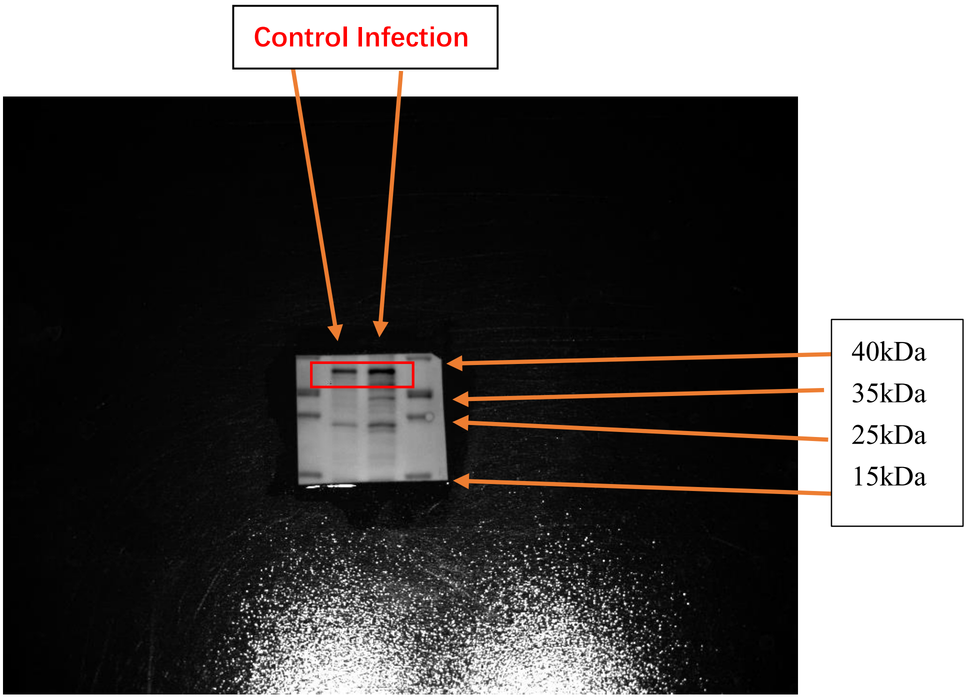

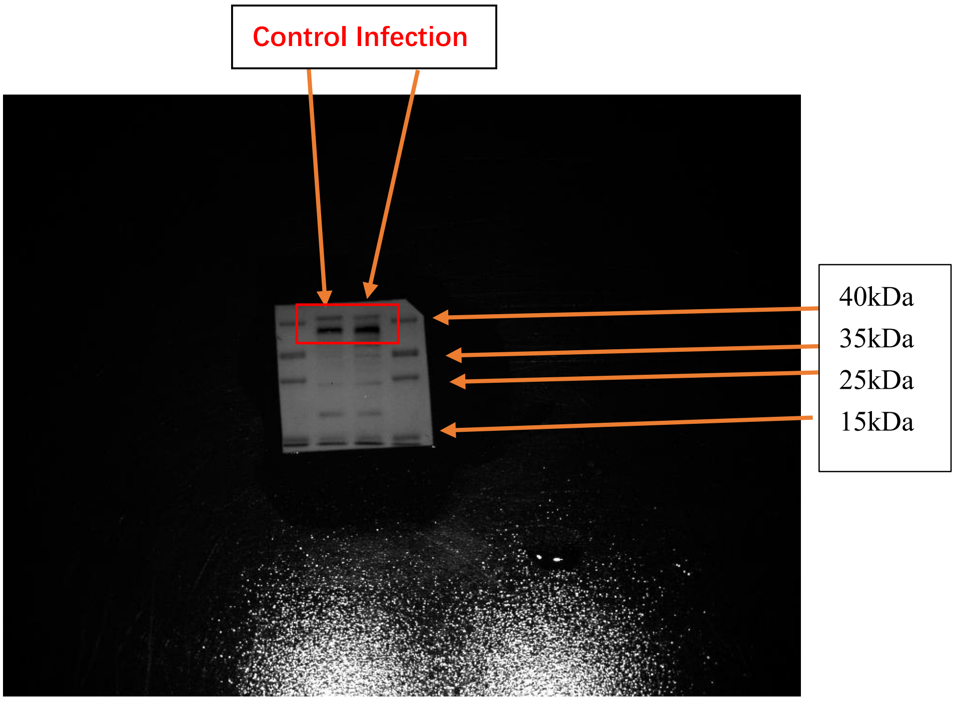
Control Infection
40kDa
35kDa
25kDa
15kDa

Fig 1.I:

GAPDH:

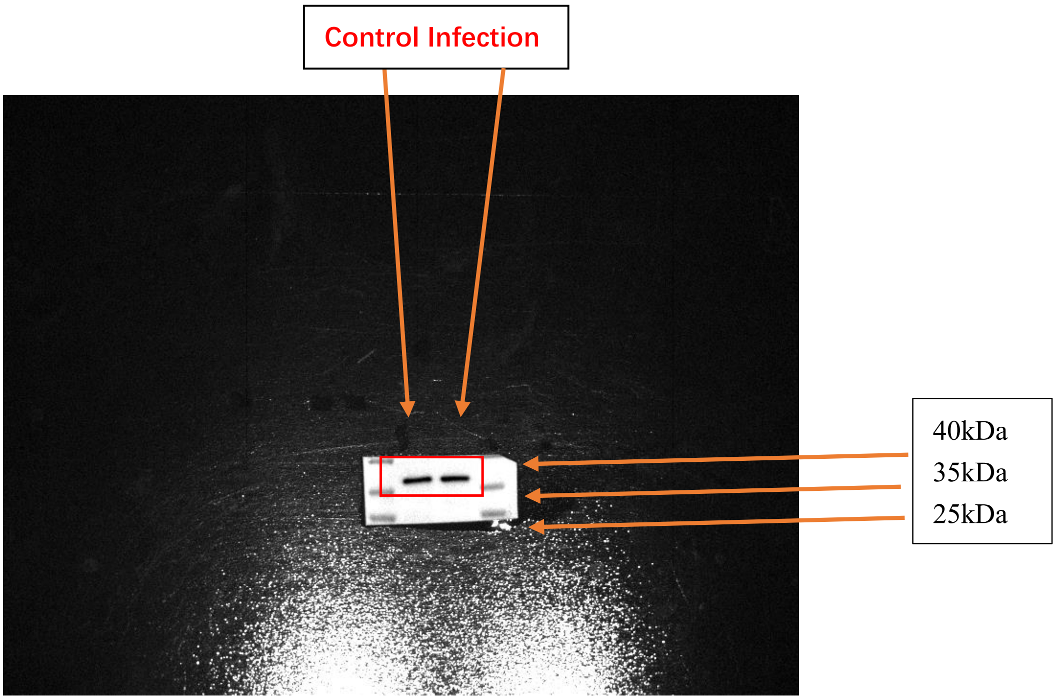
Control Infection
40kDa
35kDa
25kDa

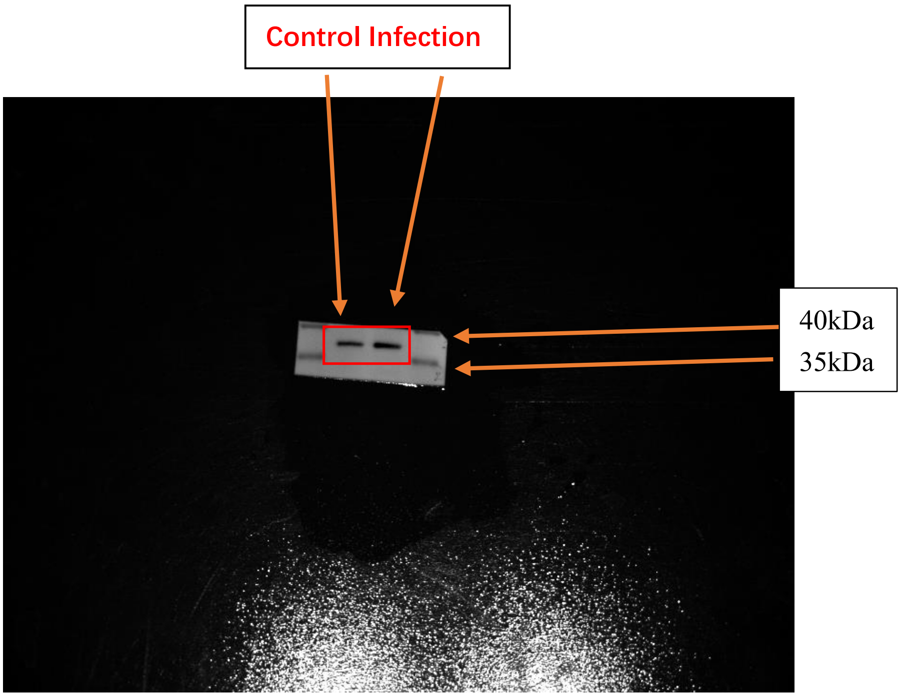
Control Infection
40kDa
35kDa

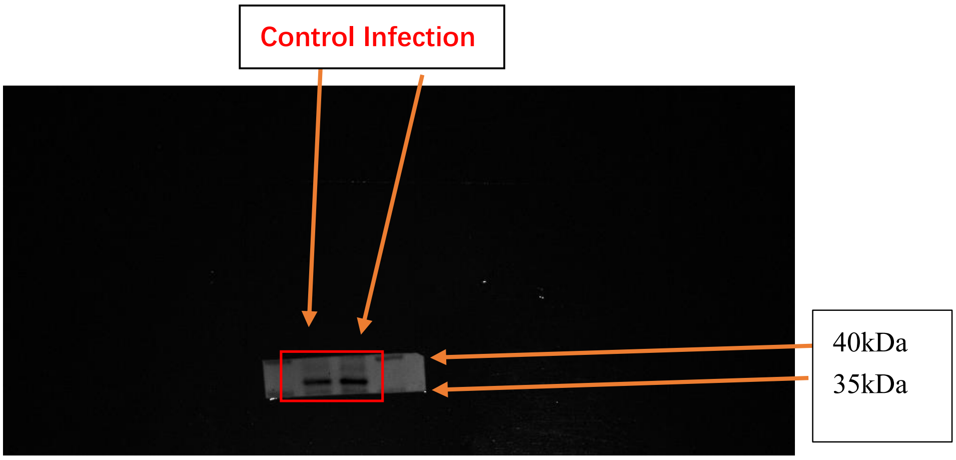
Control Infection
40kDa
35kDa

Fig 1.K:

IL-18

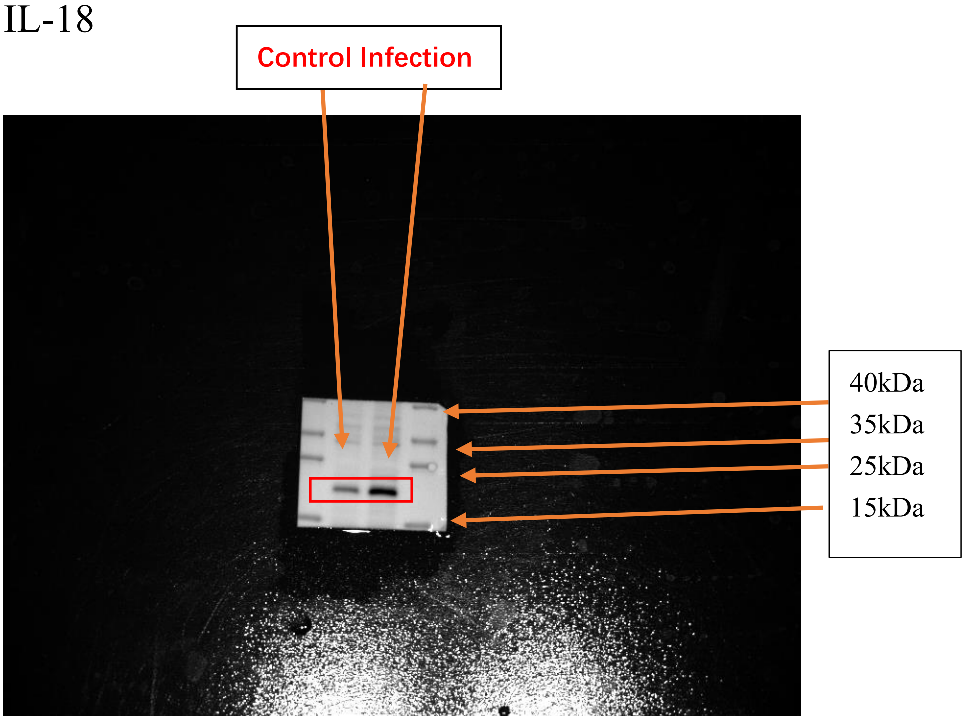
Control Infection
40kDa
35kDa
25kDa
15kDa

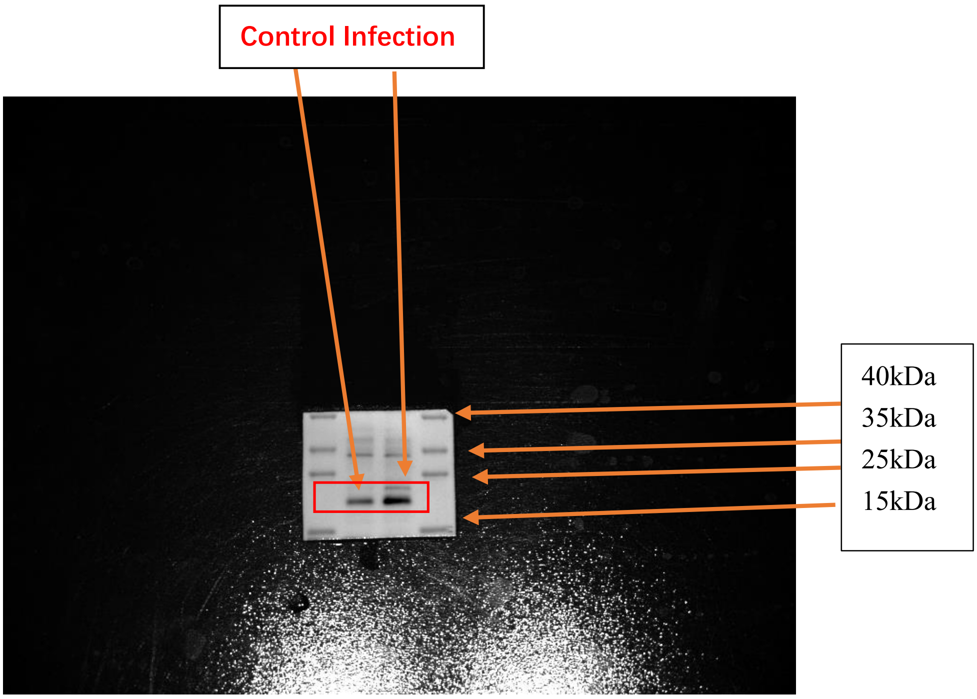
Control Infection
40kDa
35kDa
25kDa
15kDa

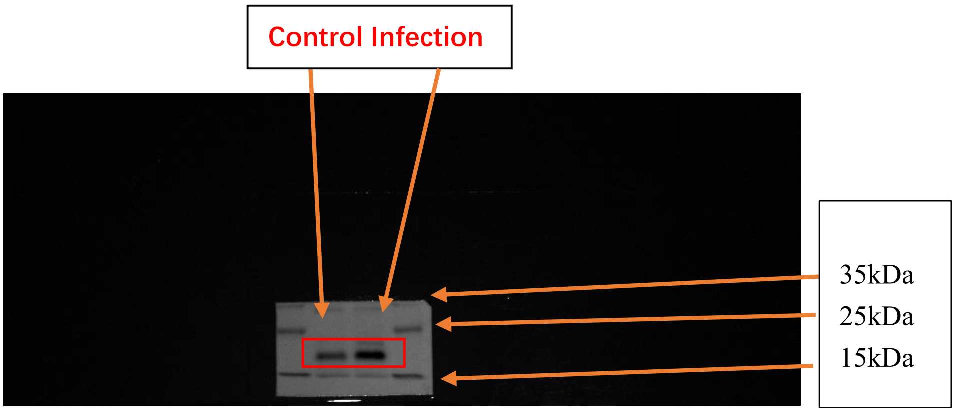
Control Infection
35kDa
25kDa
15kDa

Fig 1.K:

GAPDH

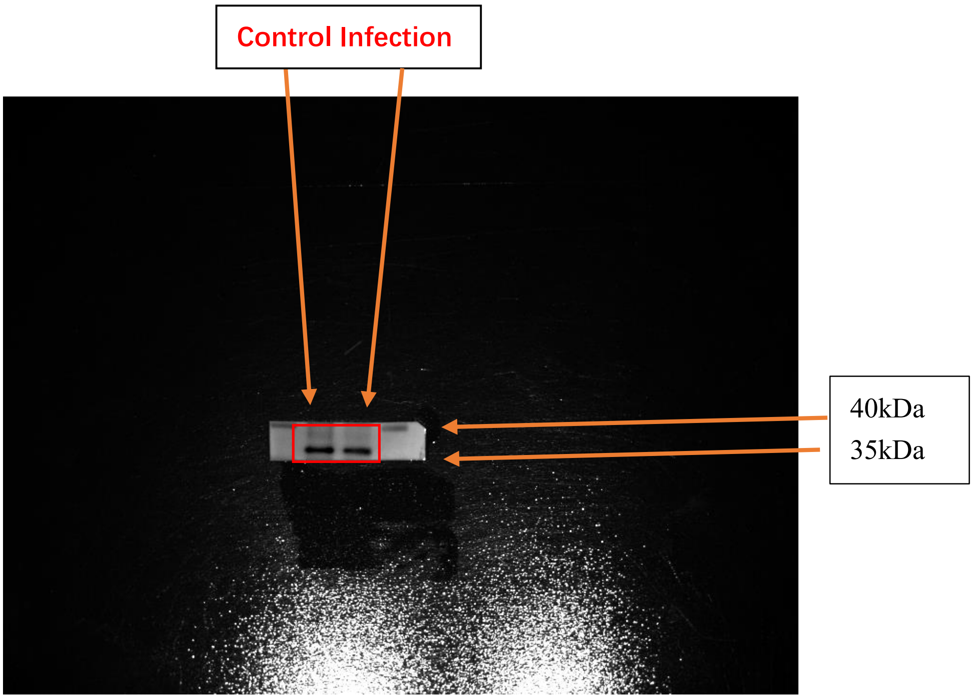
Control Infection
40kDa
35kDa

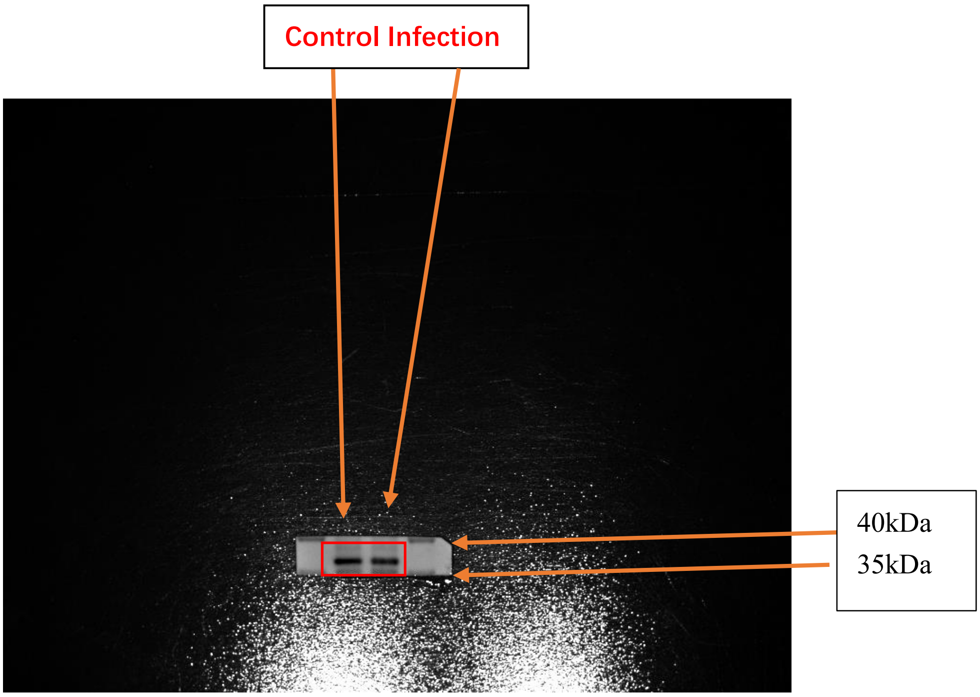
Control Infection
40kDa
35kDa

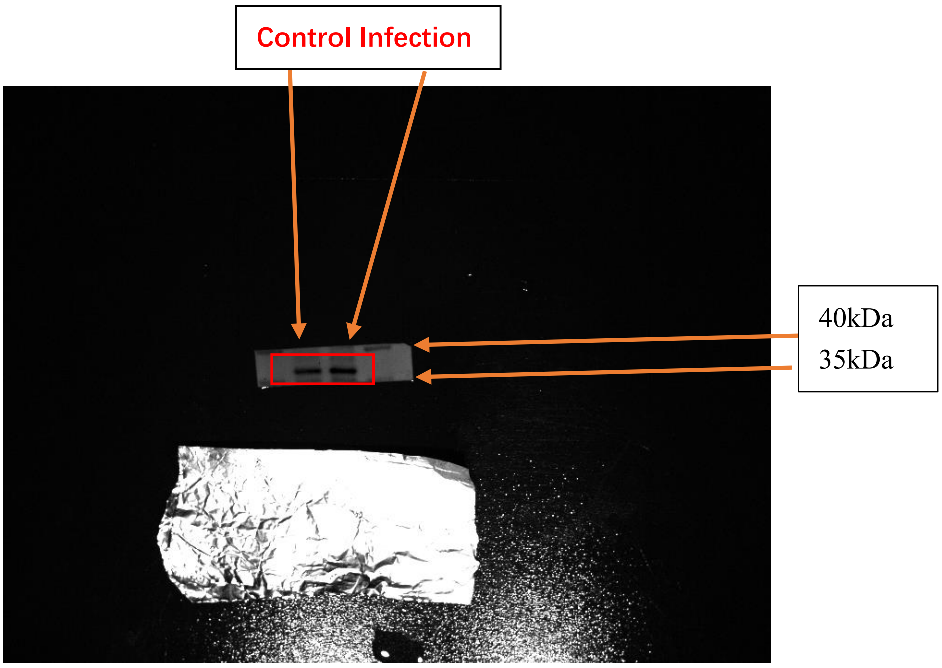
Control Infection
40kDa
35kDa

Fig 1.M:

TNF-α

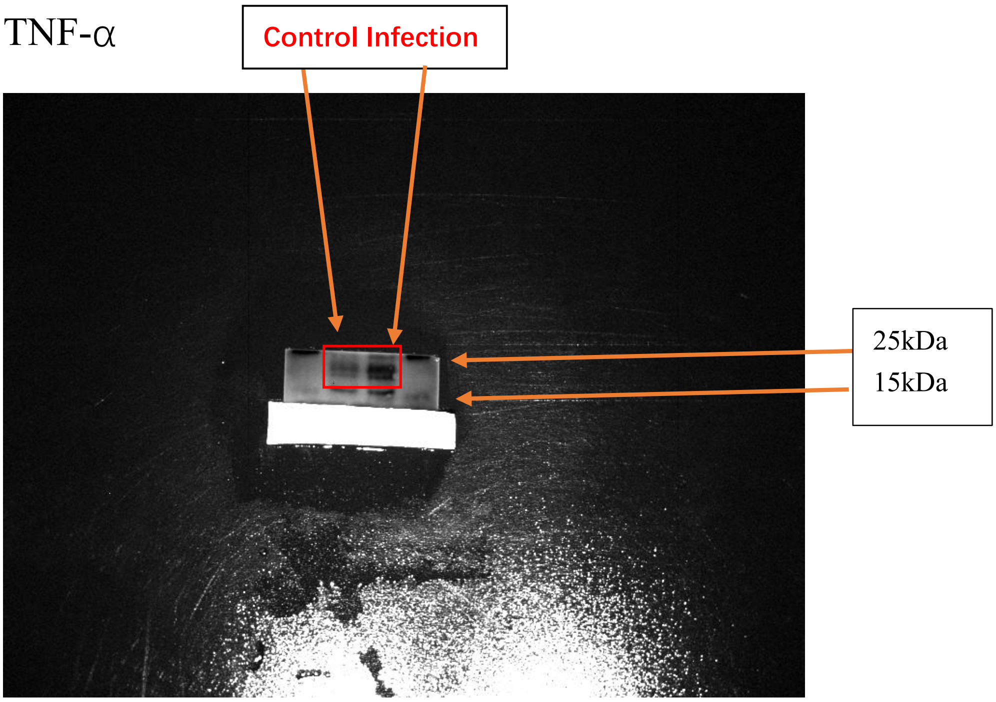
Control Infection
25kDa
15kDa

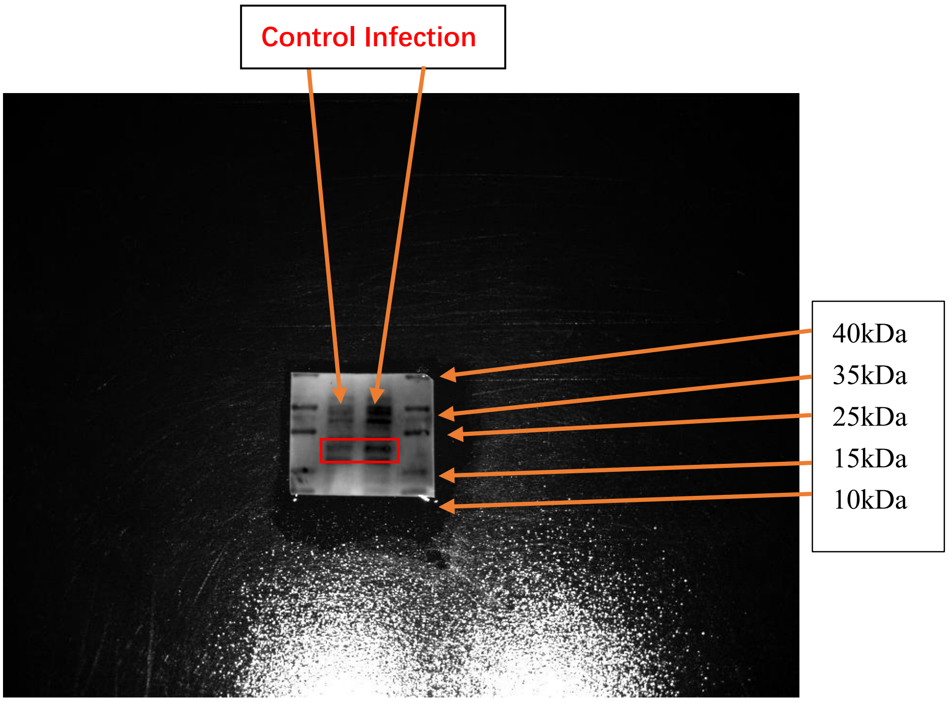
Control Infection
40kDa
35kDa
25kDa
15kDa
10kDa

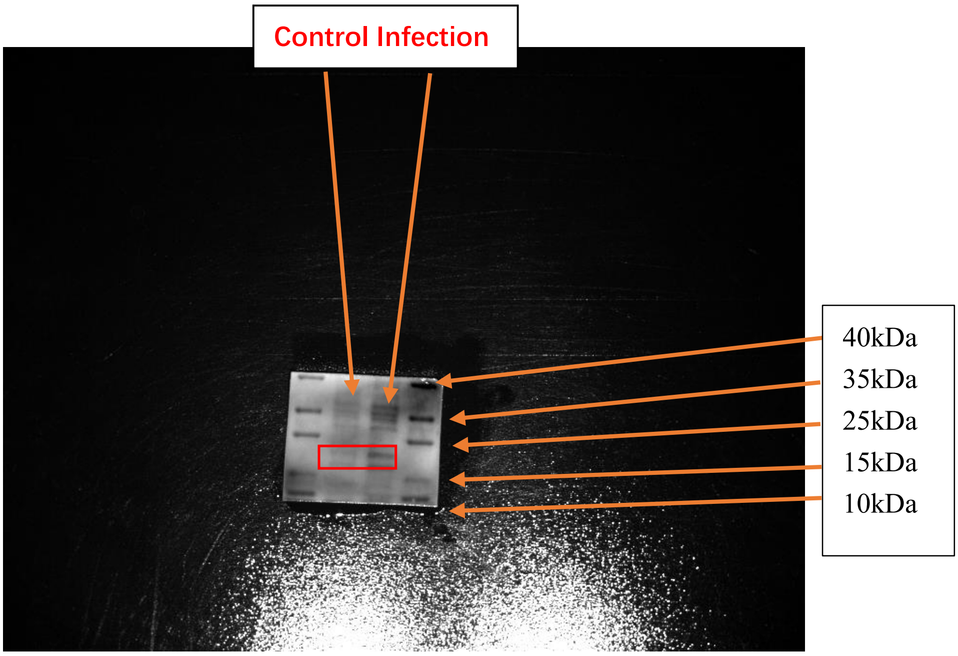
Control Infection
40kDa
35kDa
25kDa
15kDa
10kDa

Fig 1.M:

GAPDH：

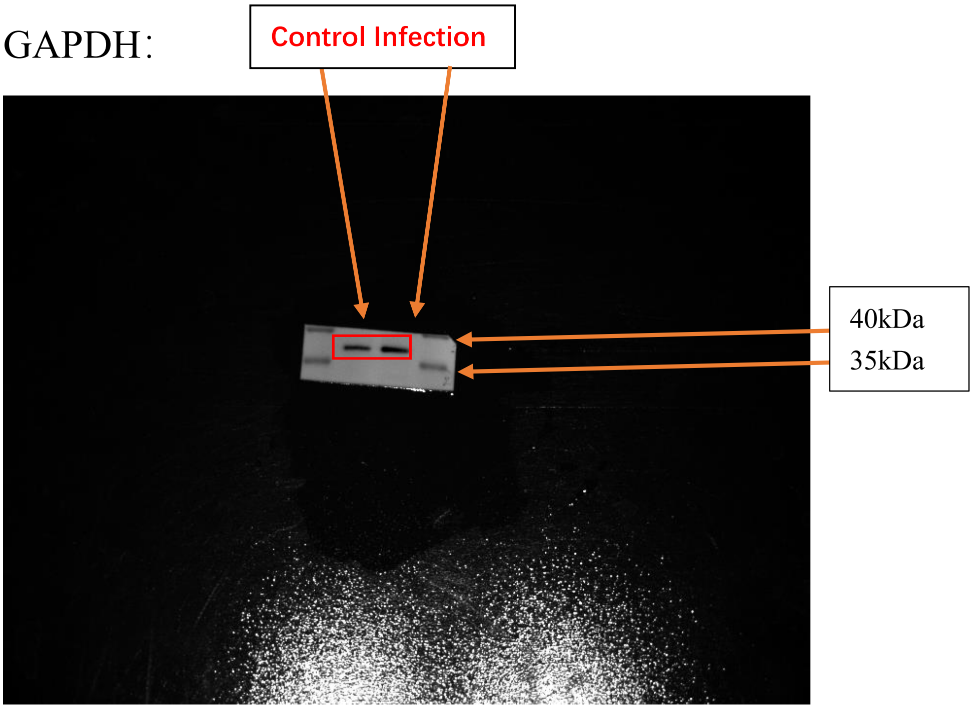
Control Infection
40kDa
35kDa

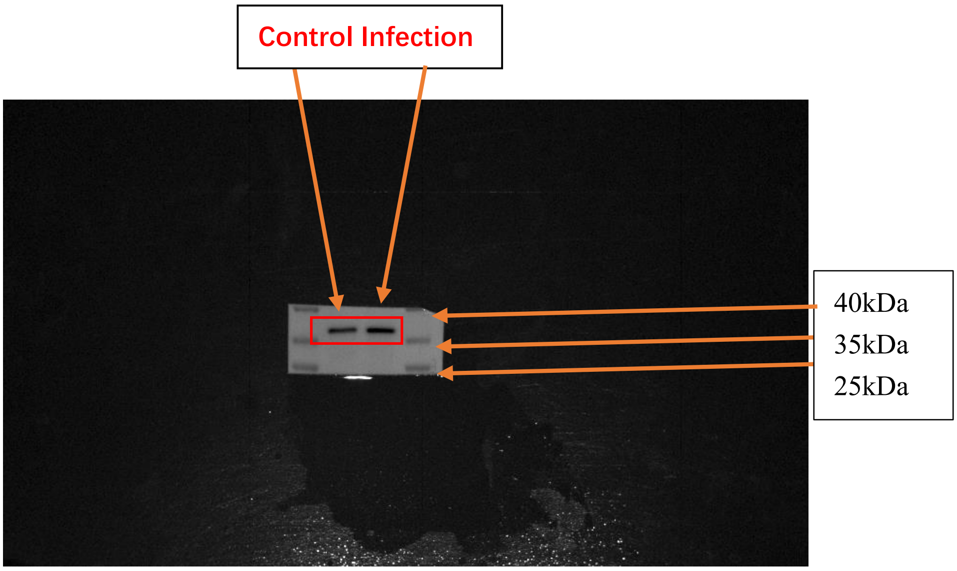
Control Infection
40kDa
35kDa
25kDa

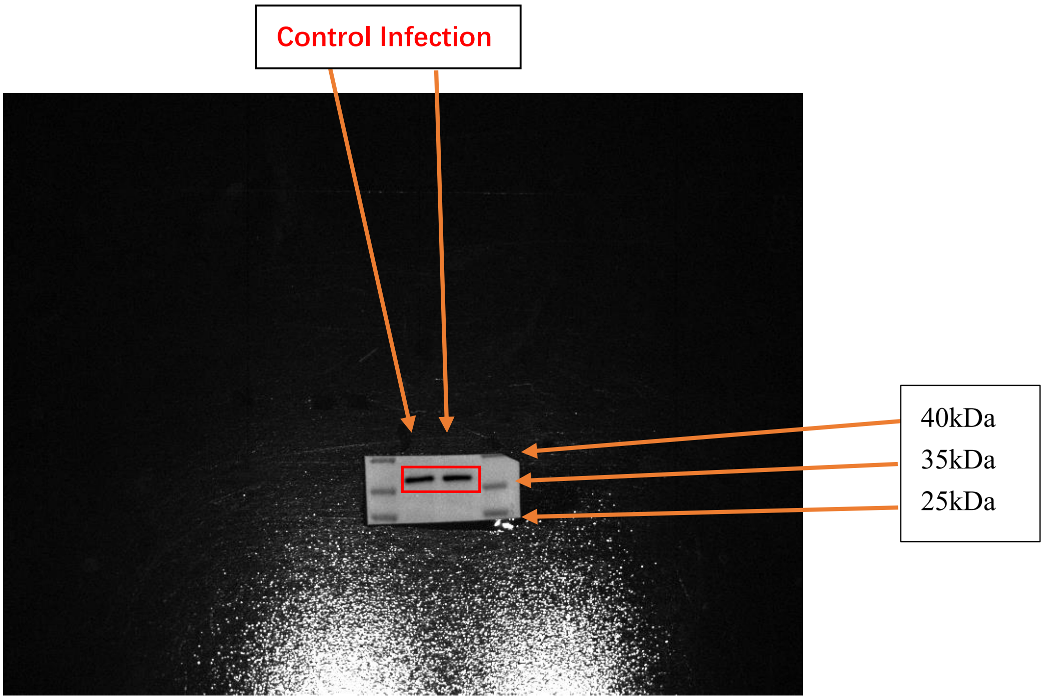
Control Infection
40kDa
35kDa
25kDa

Fig 2

Fig 2.C:

PD-1

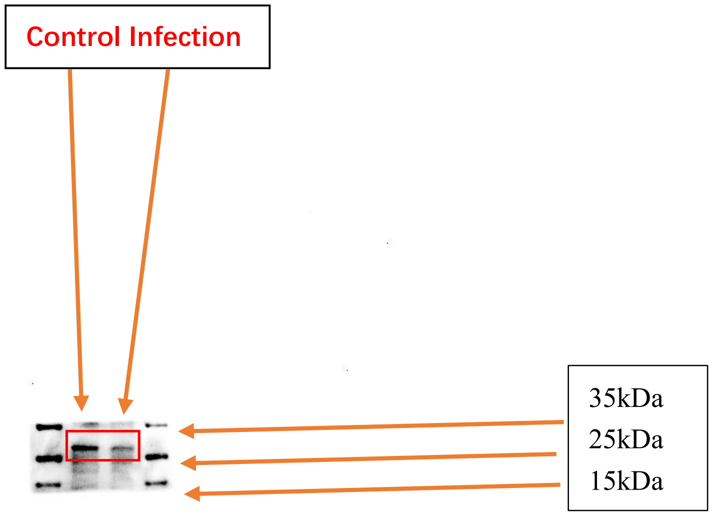

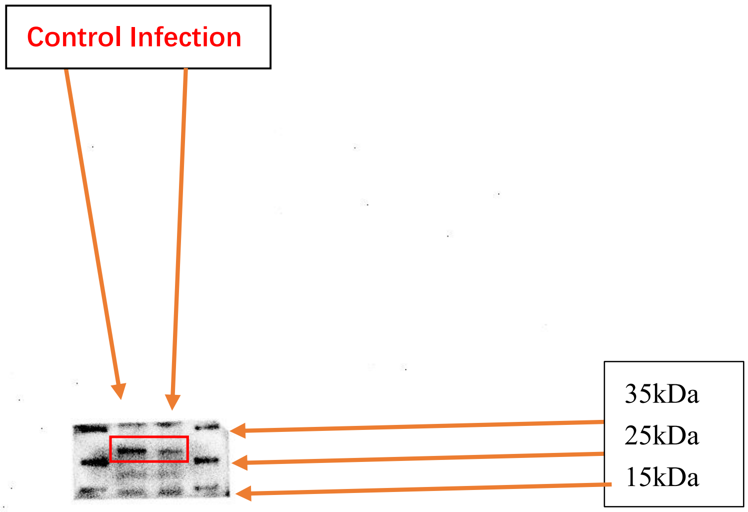

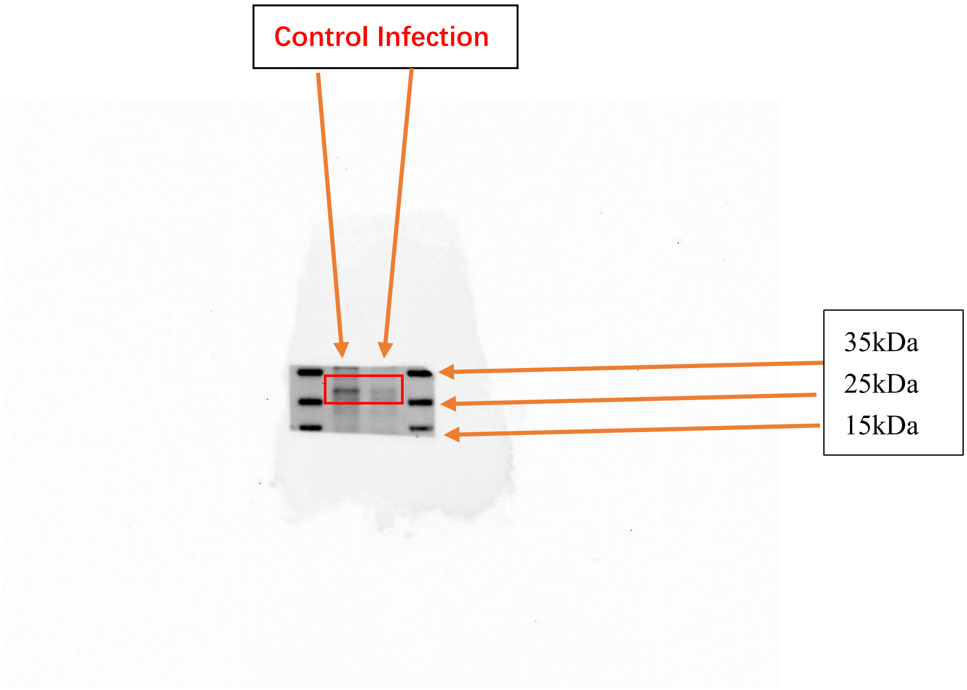
Control Infection
35kDa
25kDa
15kDa

Fig 2.C:

GAPDH

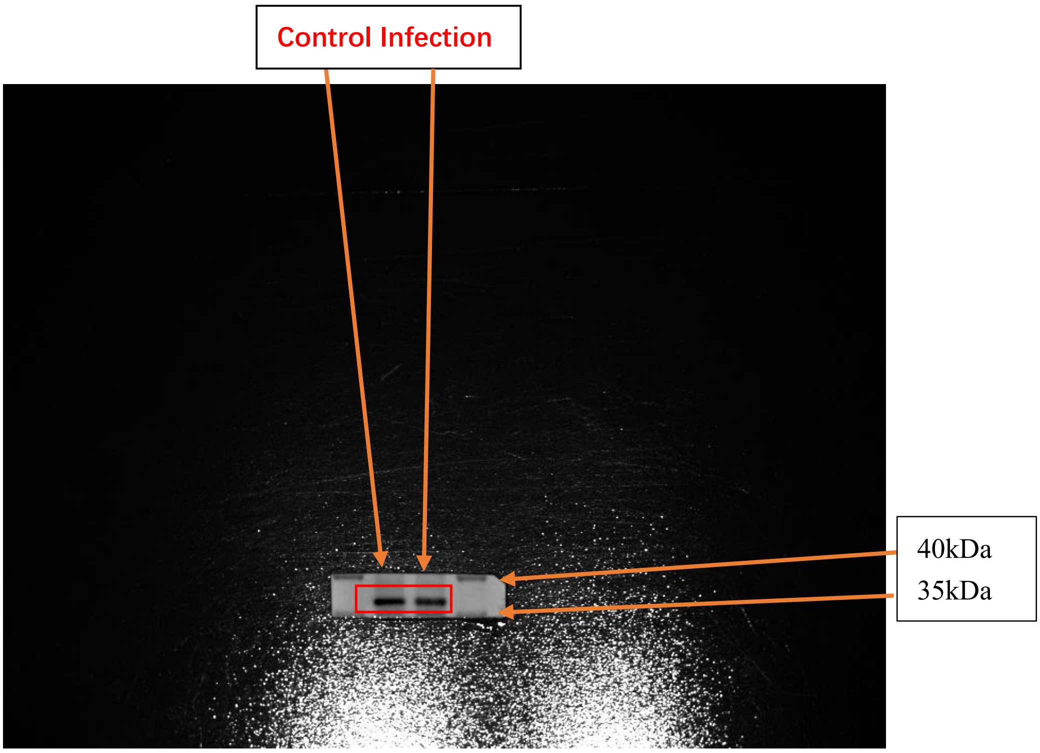
Control Infection
40kDa
35kDa

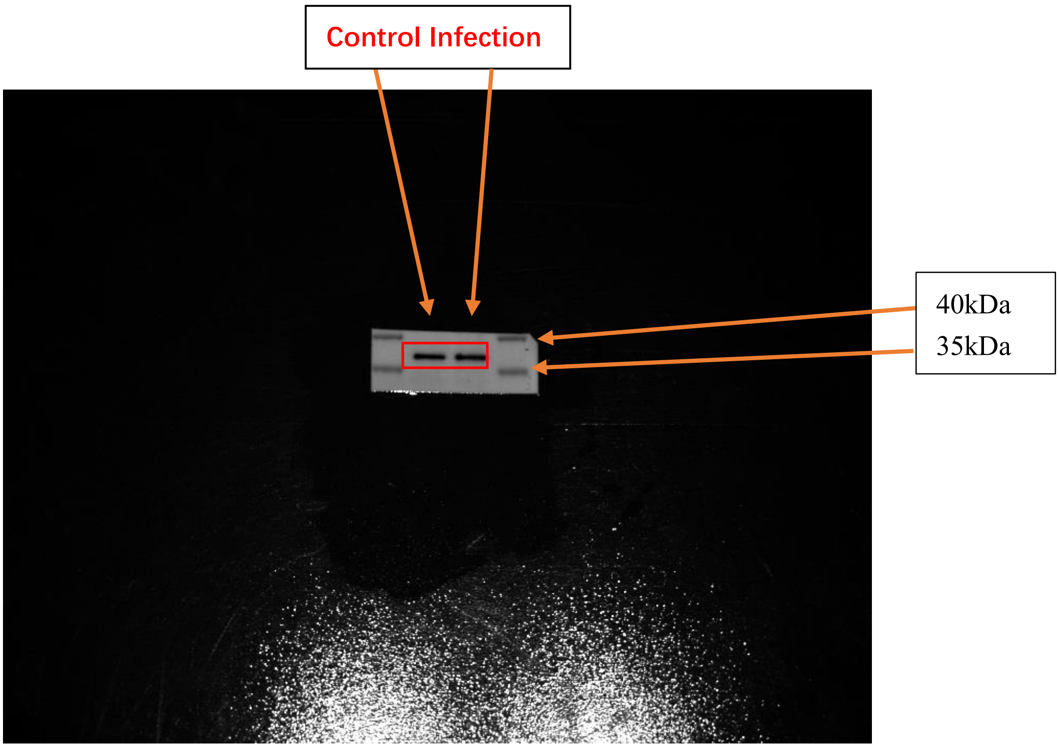
Control Infection
40kDa
35kDa

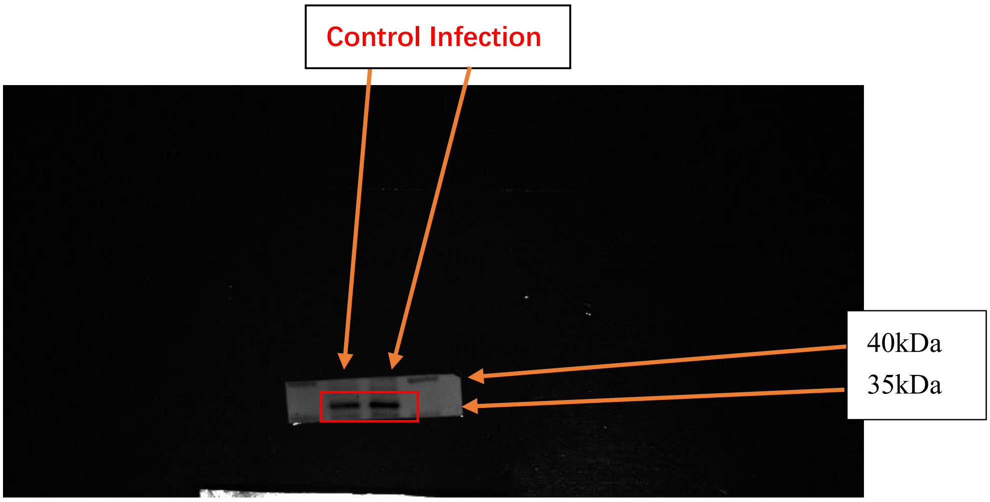
Control Infection
40kDa
35kDa

Fig 2.D:

PD-L1

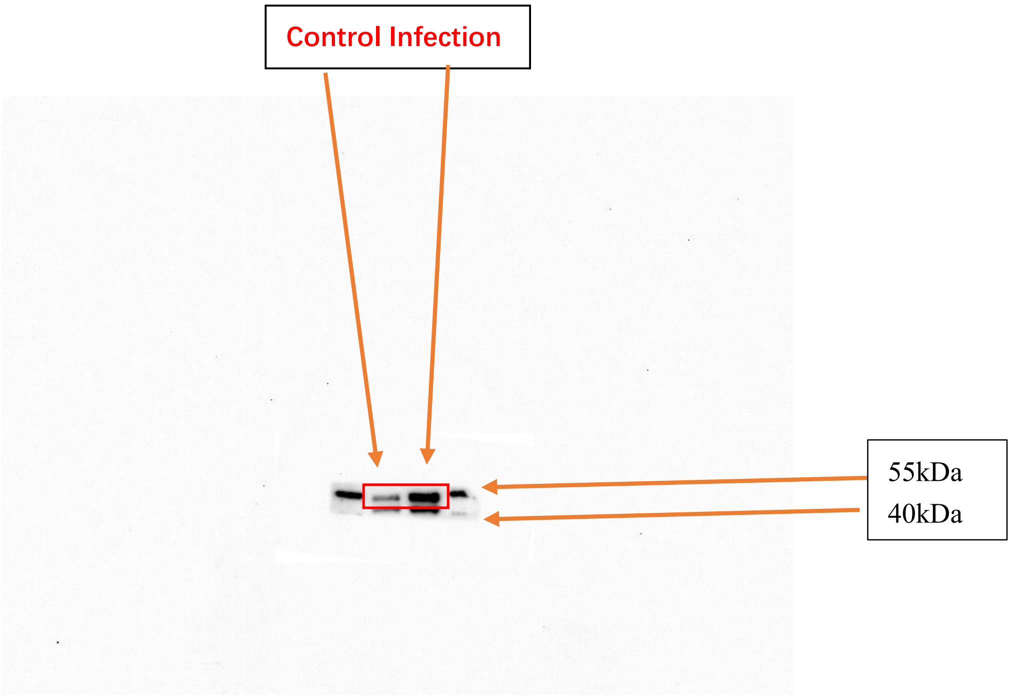
Control Infection
55kDa
40kDa

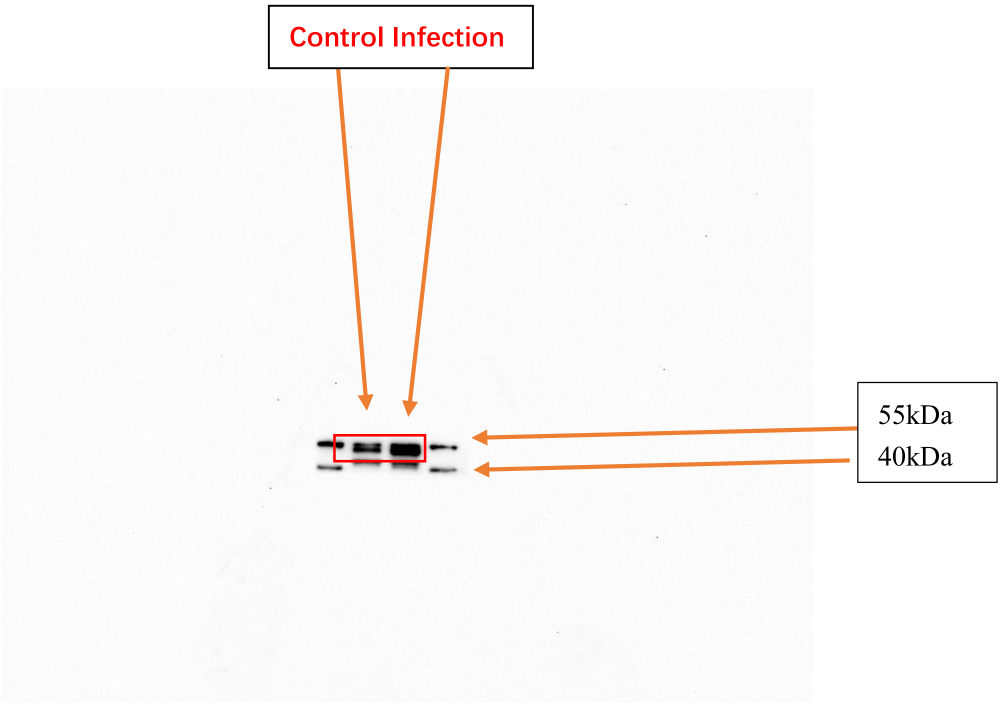
Control Infection
55kDa
40kDa

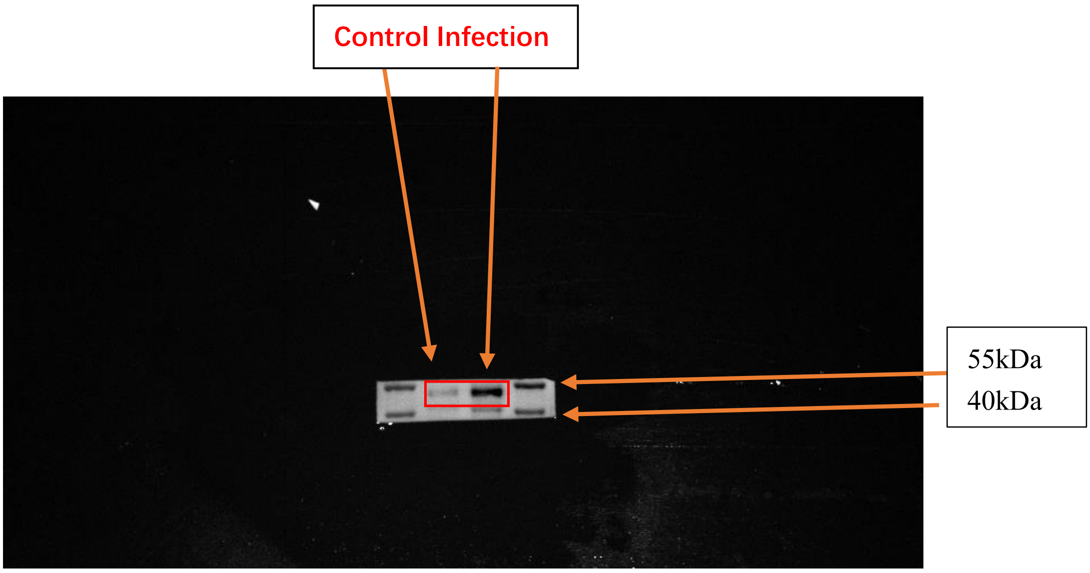
Control Infection
55kDa
40kDa

Fig 2.D:

GAPDH

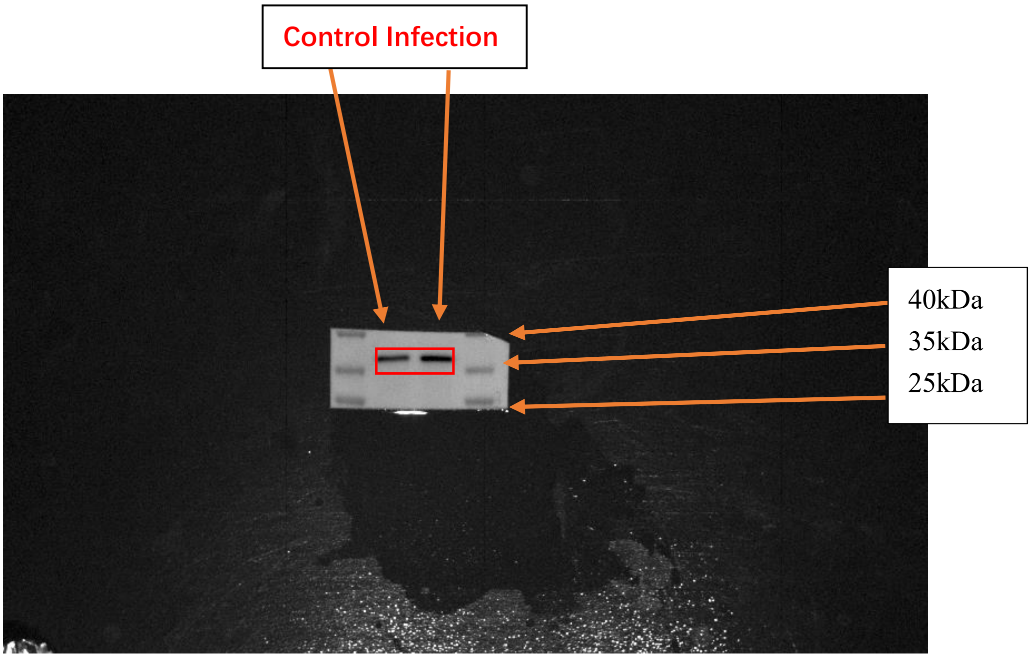
Control Infection
40kDa
35kDa
25kDa

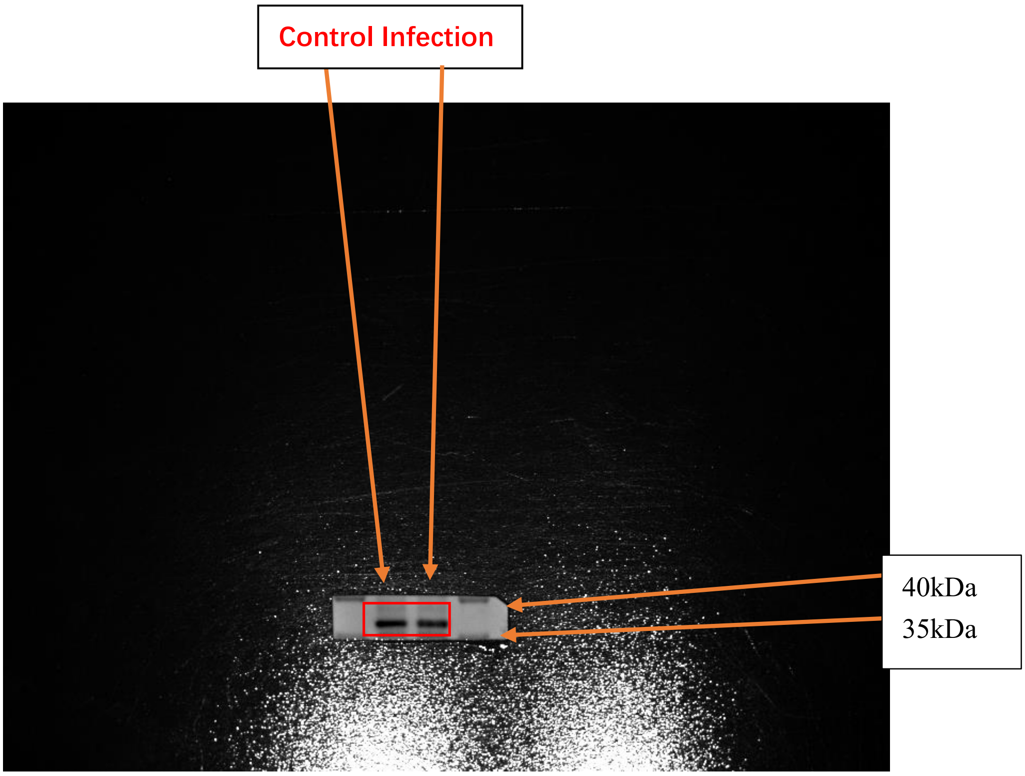
Control Infection
40kDa
35kDa

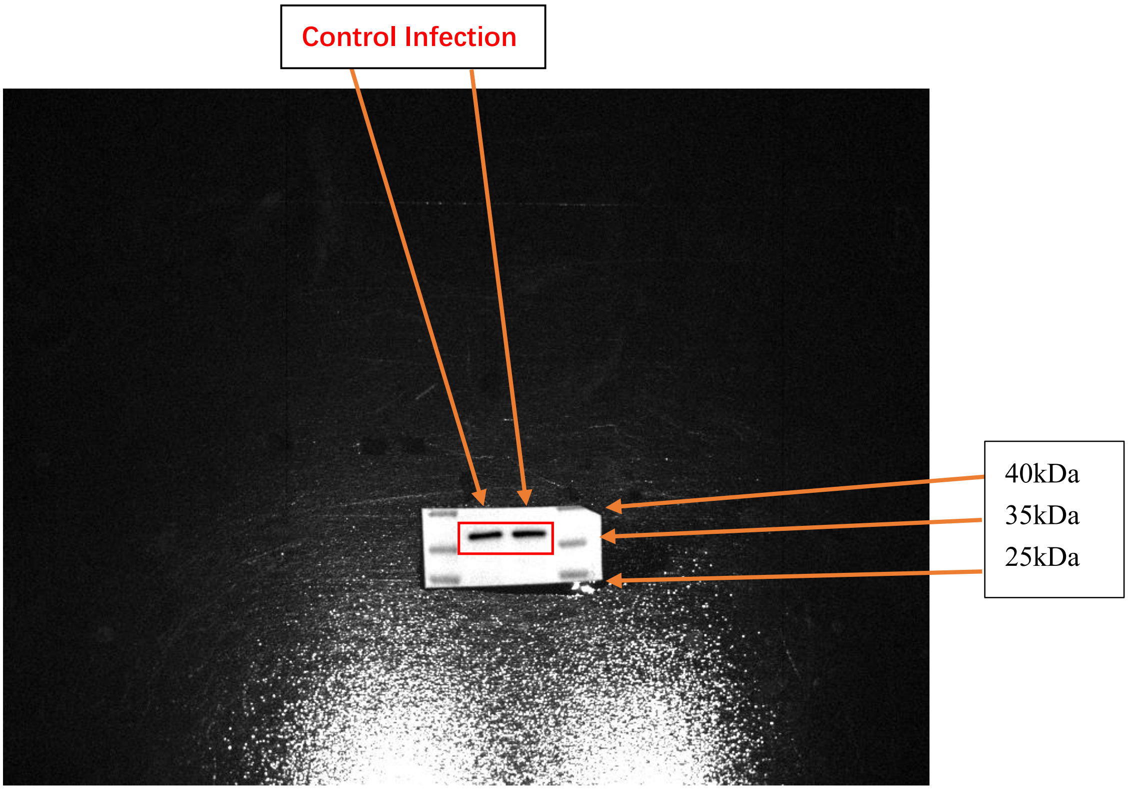
Control Infection
40kDa
35kDa
25kDa

Fig 7

Fig 7.D

p-PI3K

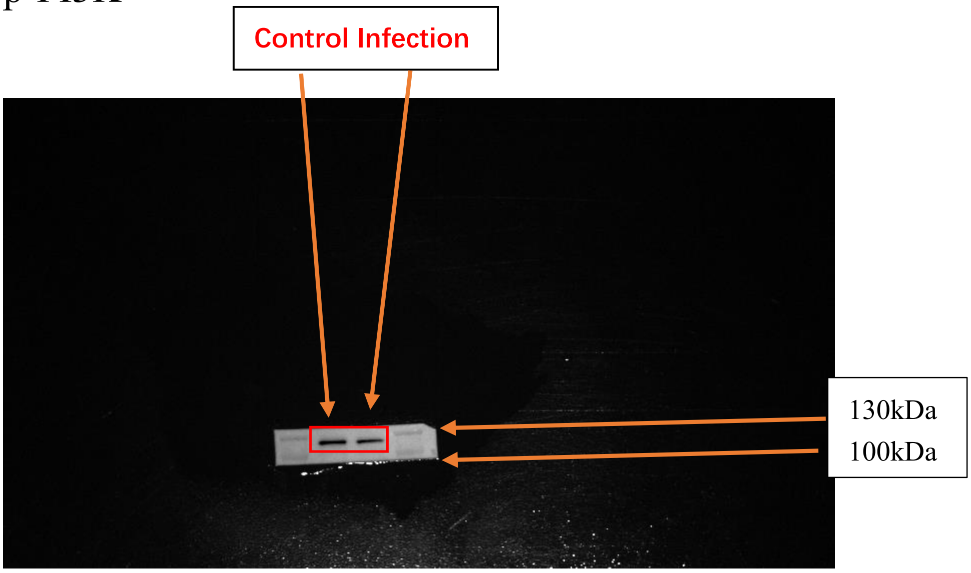

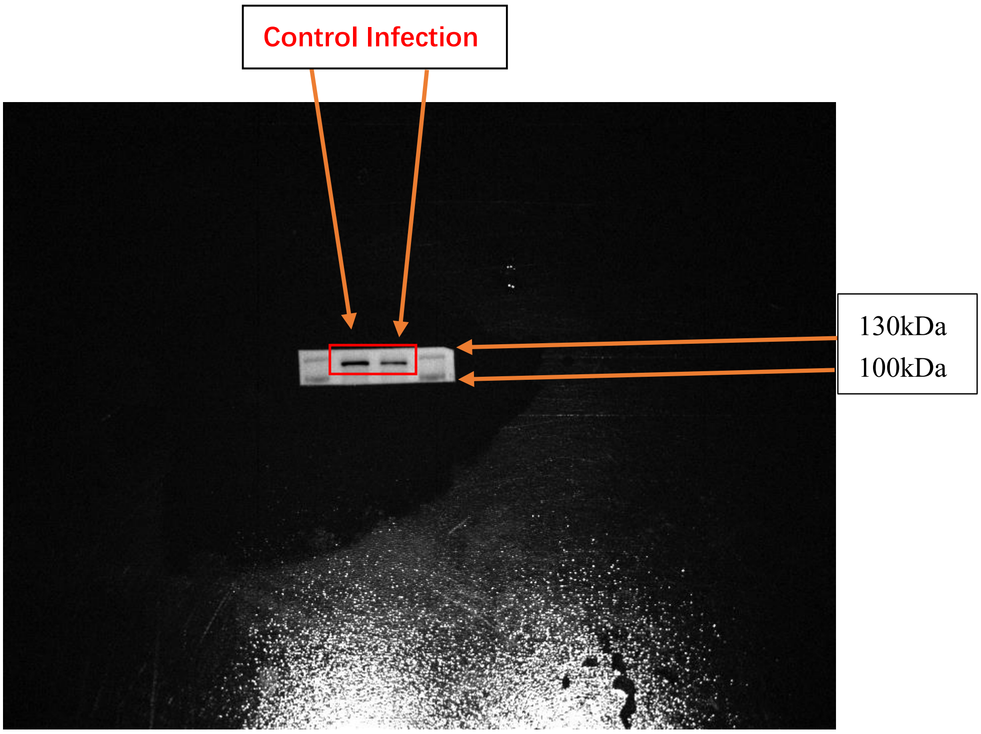

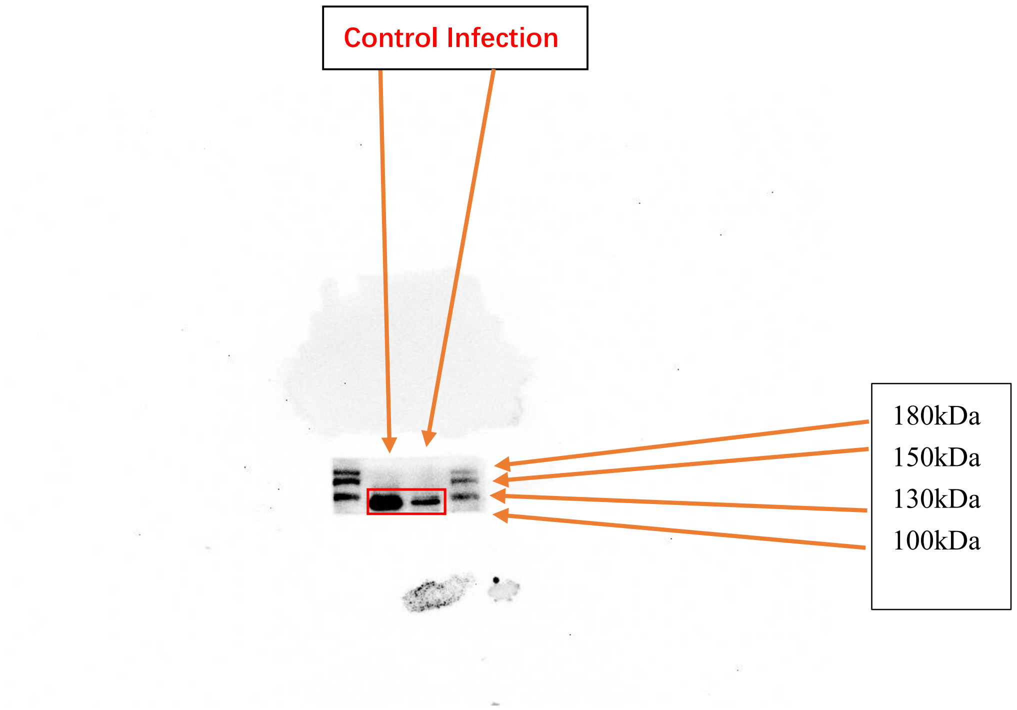
Control Infection
180kDa
150kDa
130kDa
100kDa

Fig 7.D

PI3K

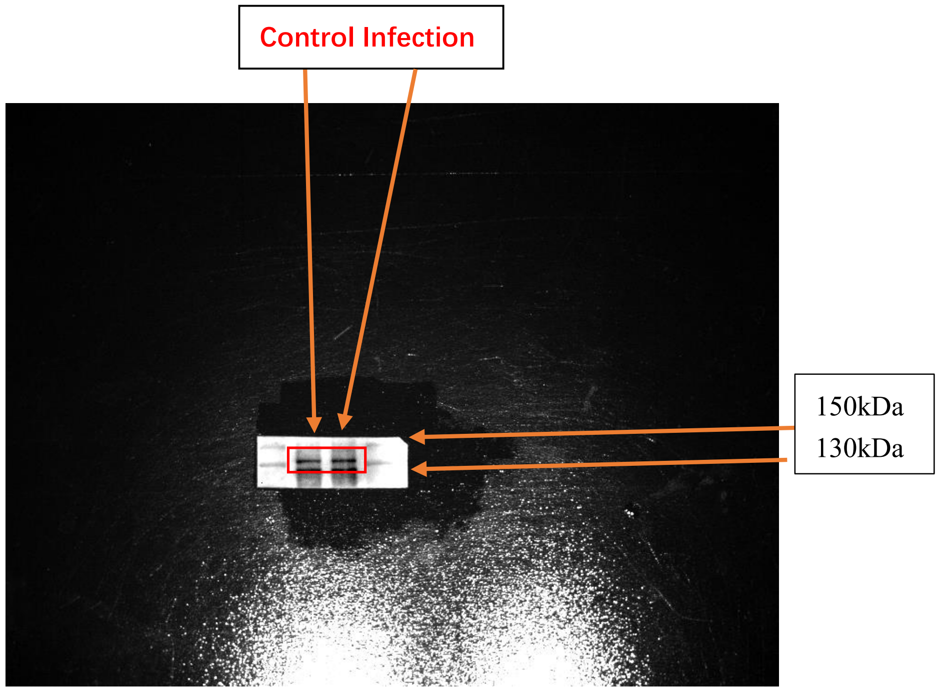
Control Infection
150kDa
130kDa

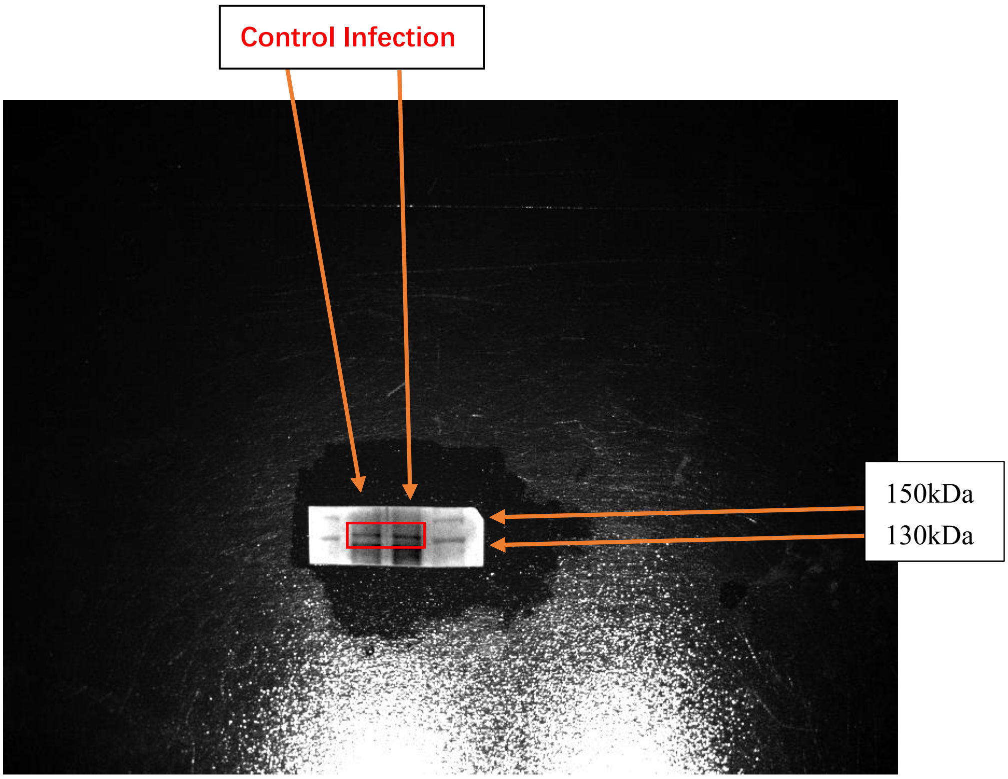
Control Infection
150kDa
130kDa

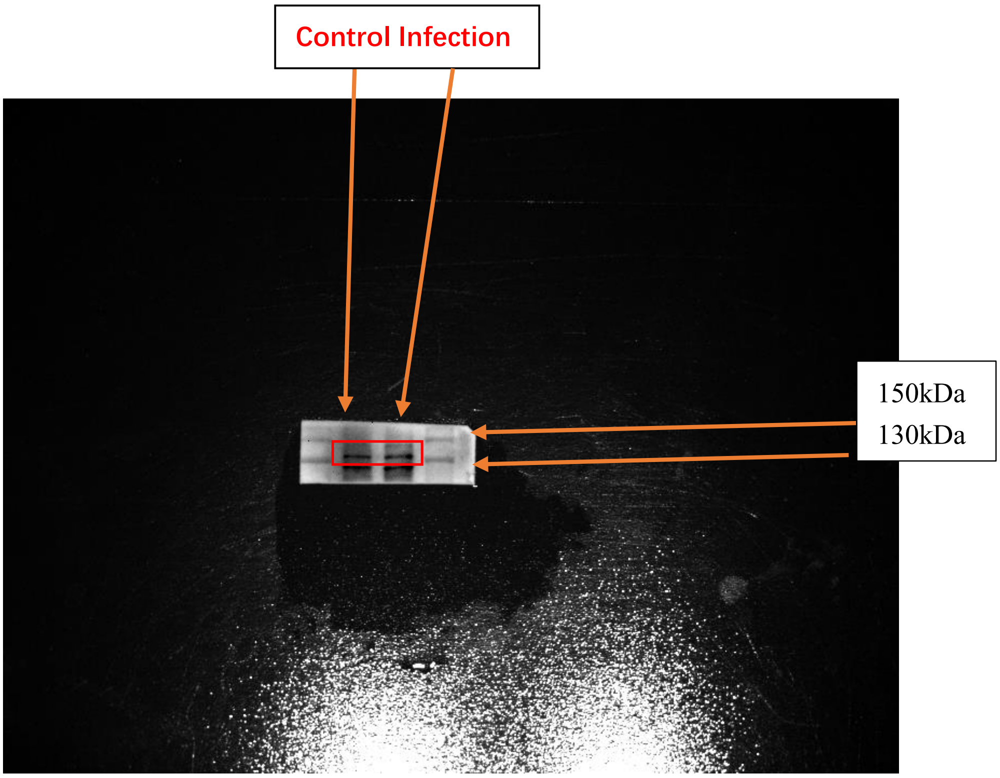
Control Infection
150kDa
130kDa

Fig 7.D

p-Akt

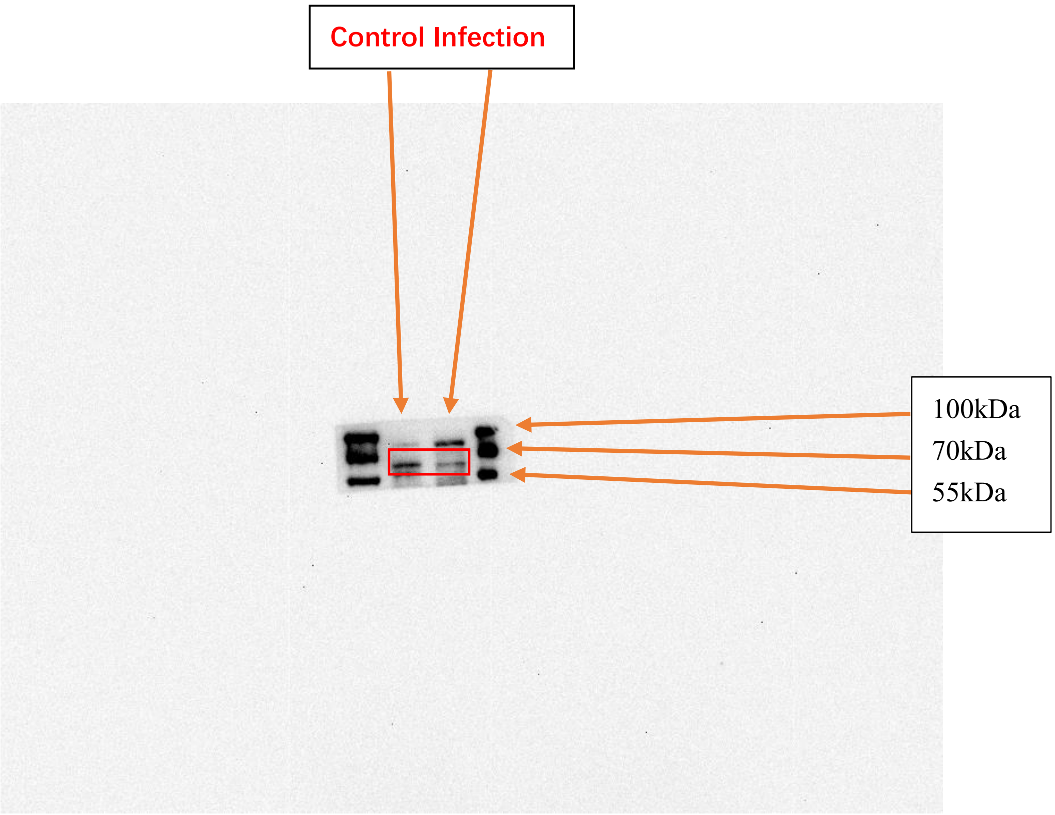
Control Infection
100kDa
70kDa
55kDa

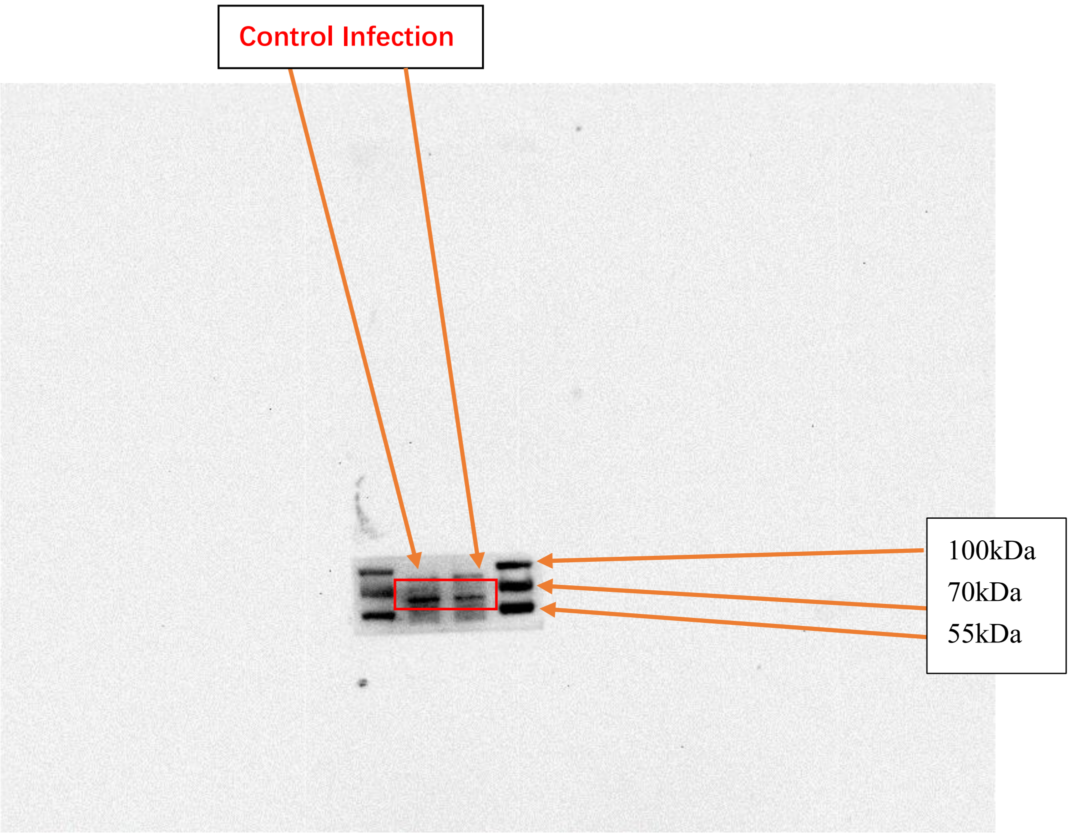
Control Infection
100kDa
70kDa
55kDa

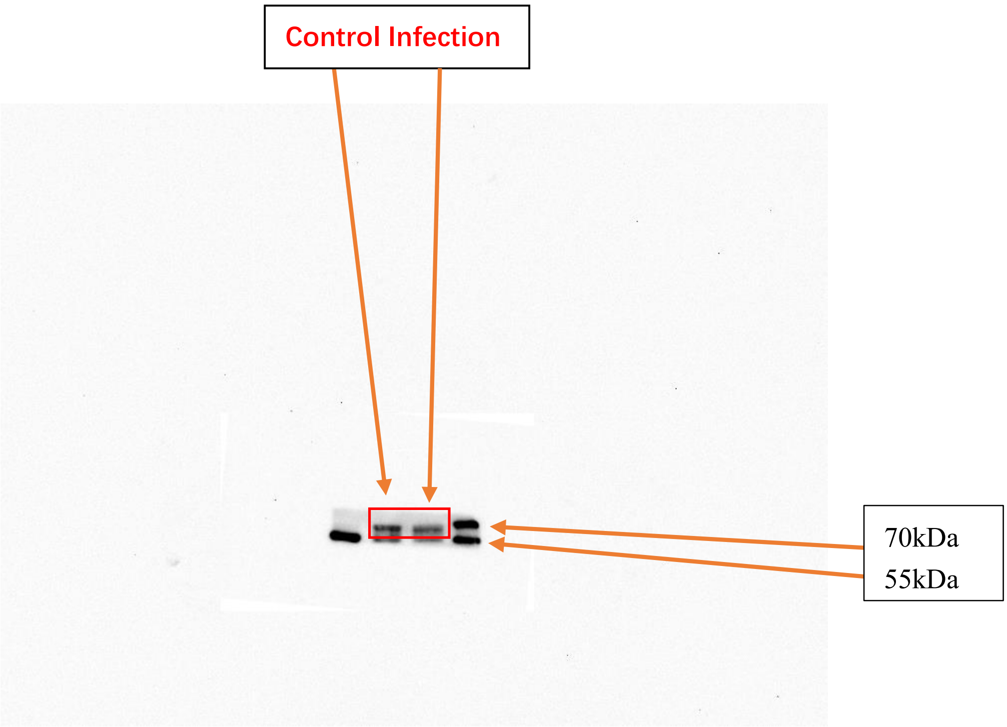
Control Infection
70kDa
55kDa

Fig 7.D

Akt

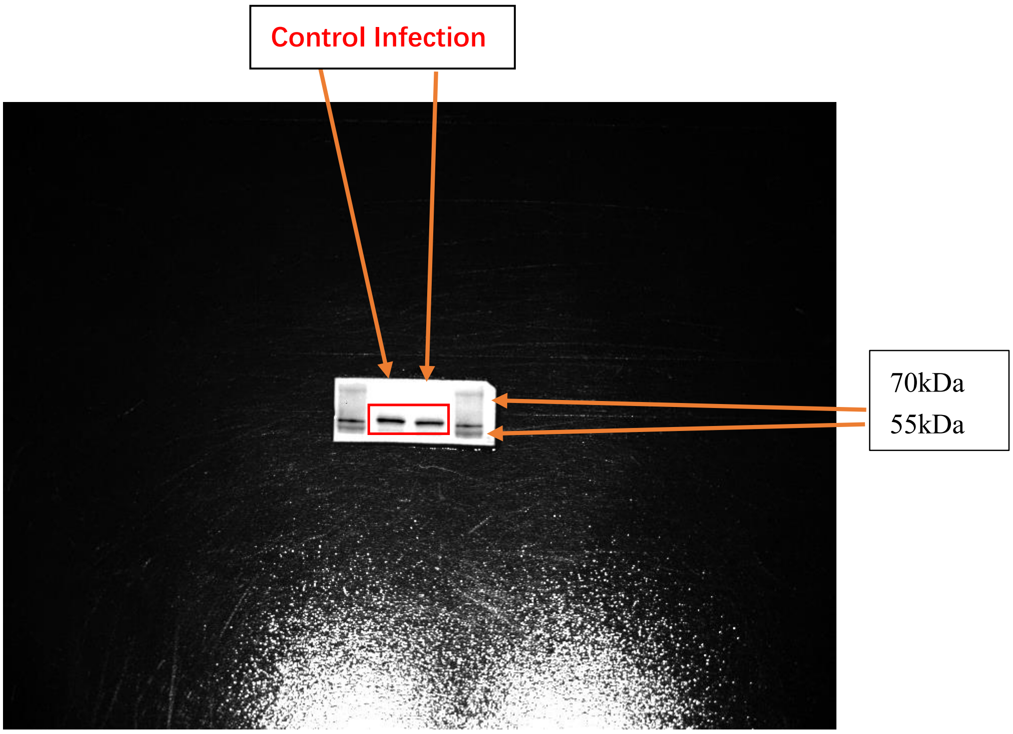
Control Infection
70kDa
55kDa

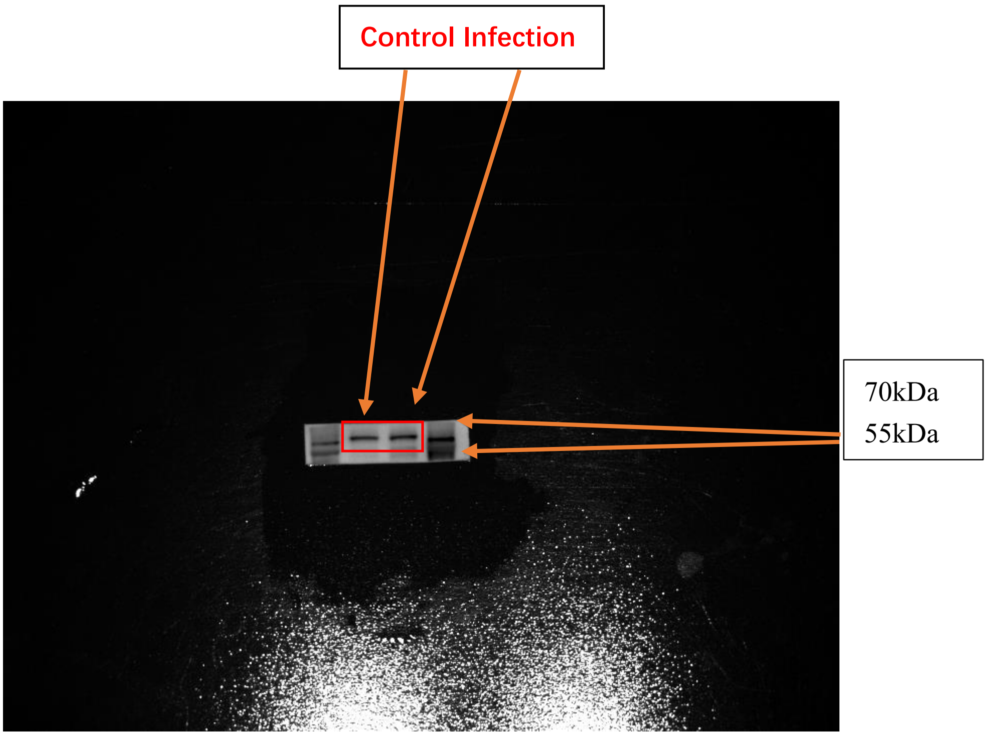
Control Infection
70kDa
55kDa

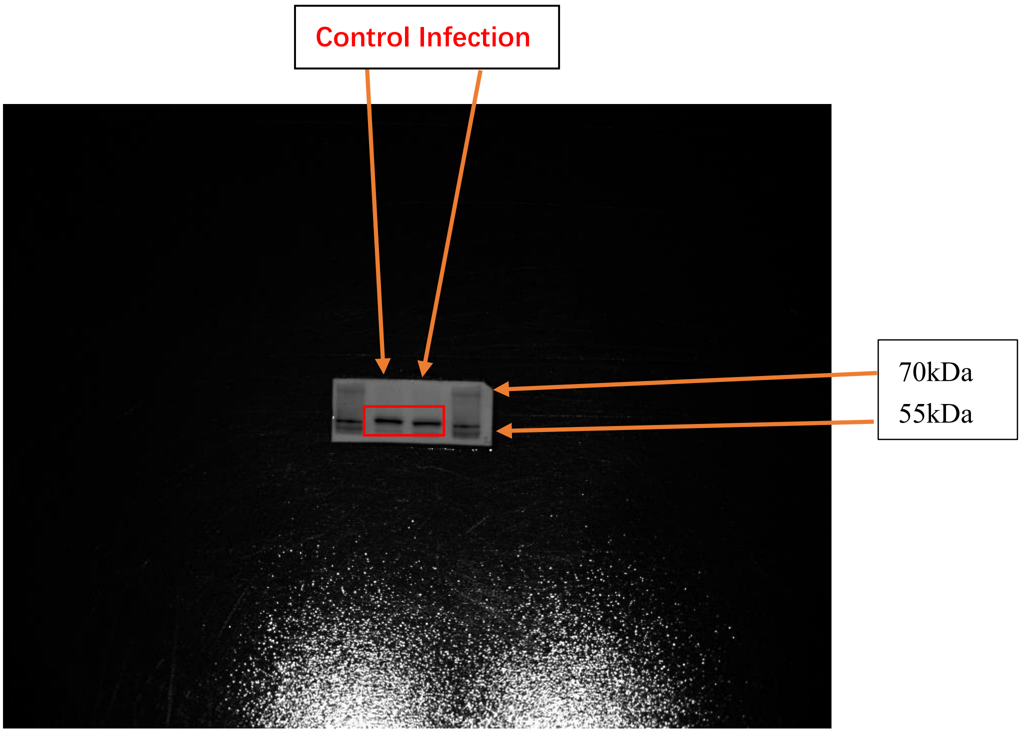
Control Infection
70kDa
55kDa

Fig 7.D

p-mTOR

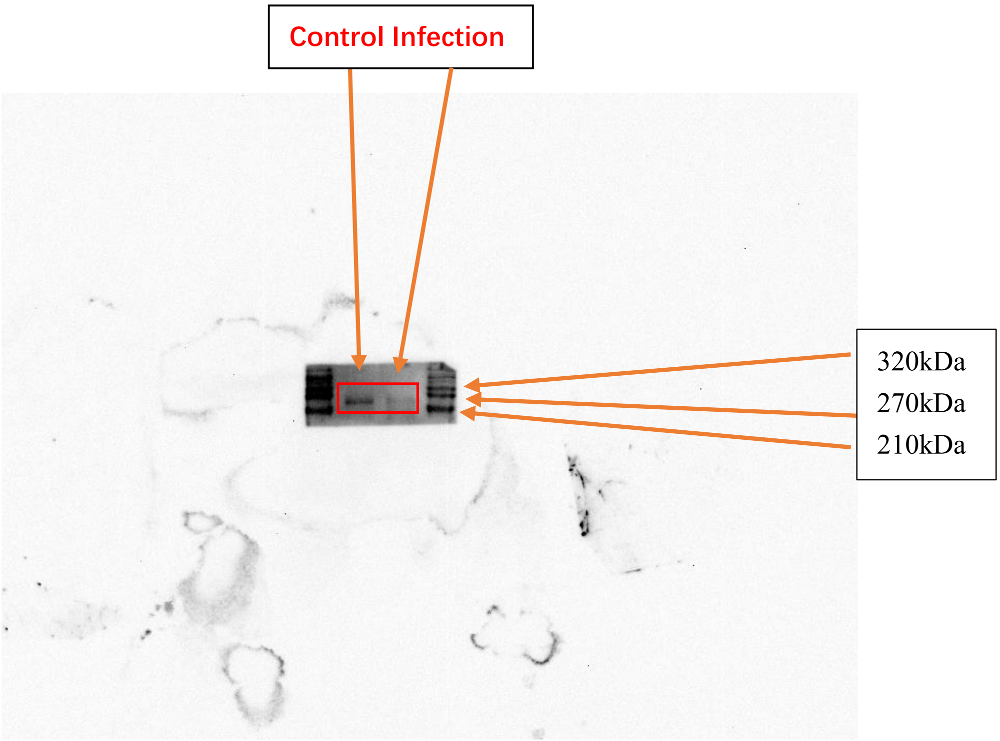
Control Infection
320kDa
270kDa
210kDa

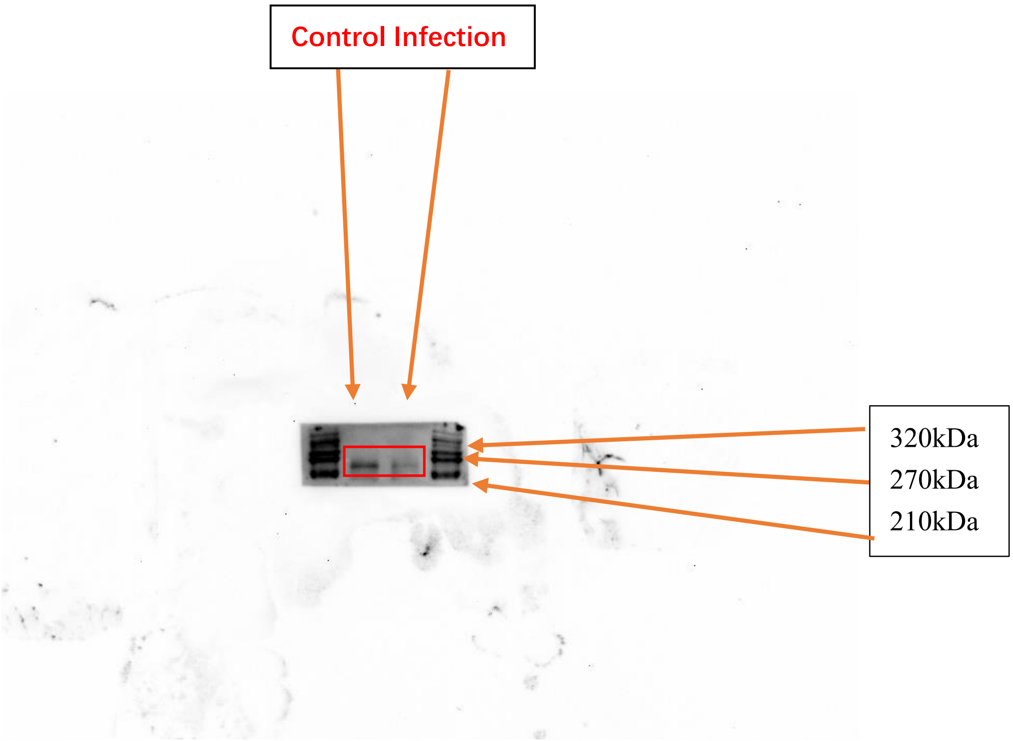
Control Infection
320kDa
270kDa
210kDa

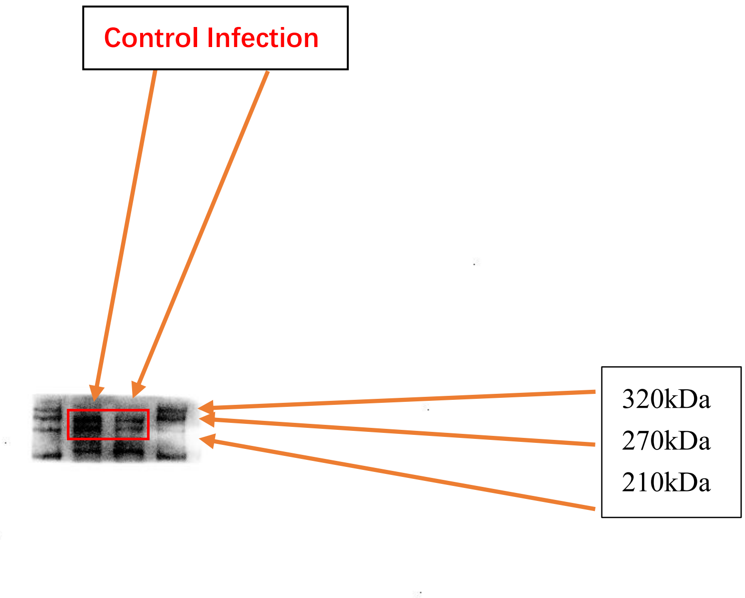
Control Infection
320kDa
270kDa
210kDa

Fig 7.D

mTOR

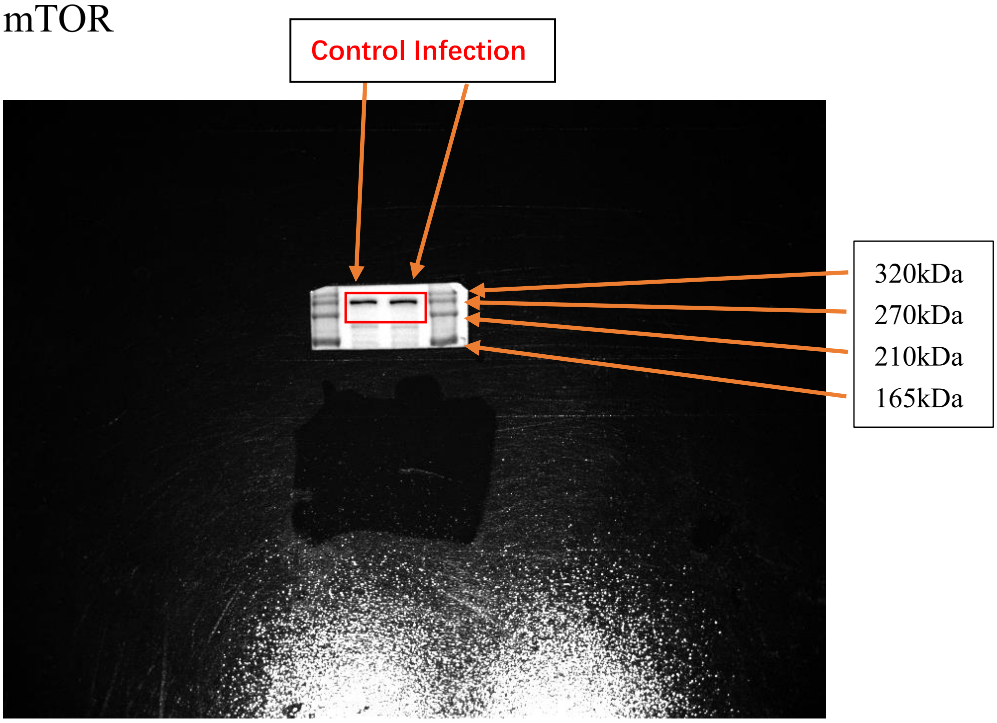
Control Infection
320kDa
270kDa
210kDa
165kDa

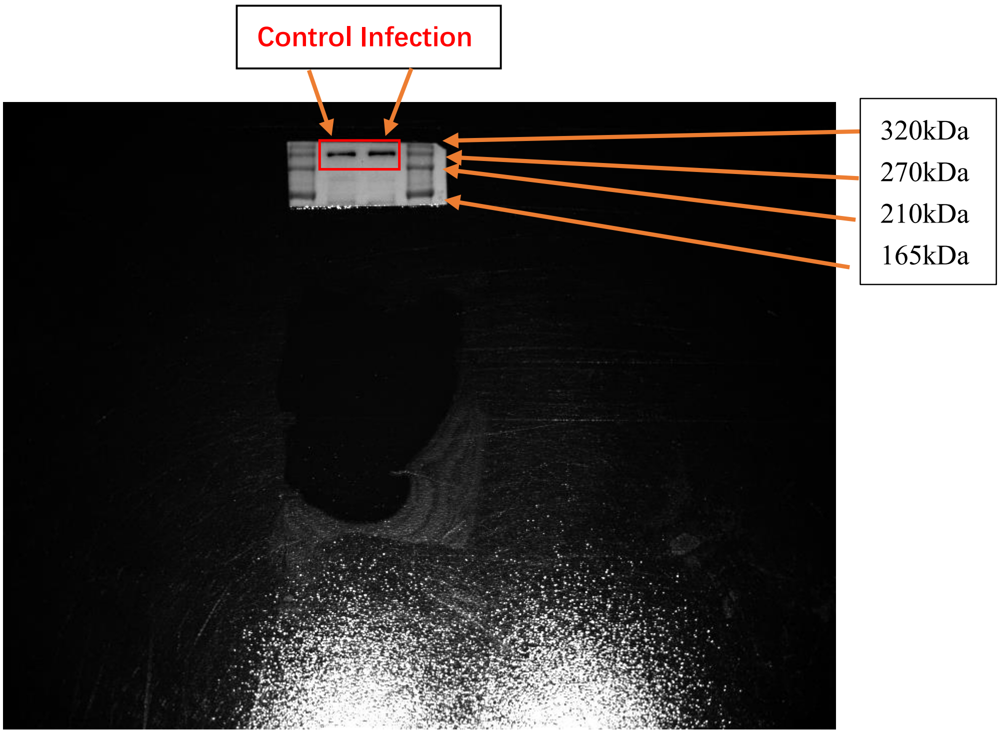
Control Infection
320kDa
270kDa
210kDa
165kDa

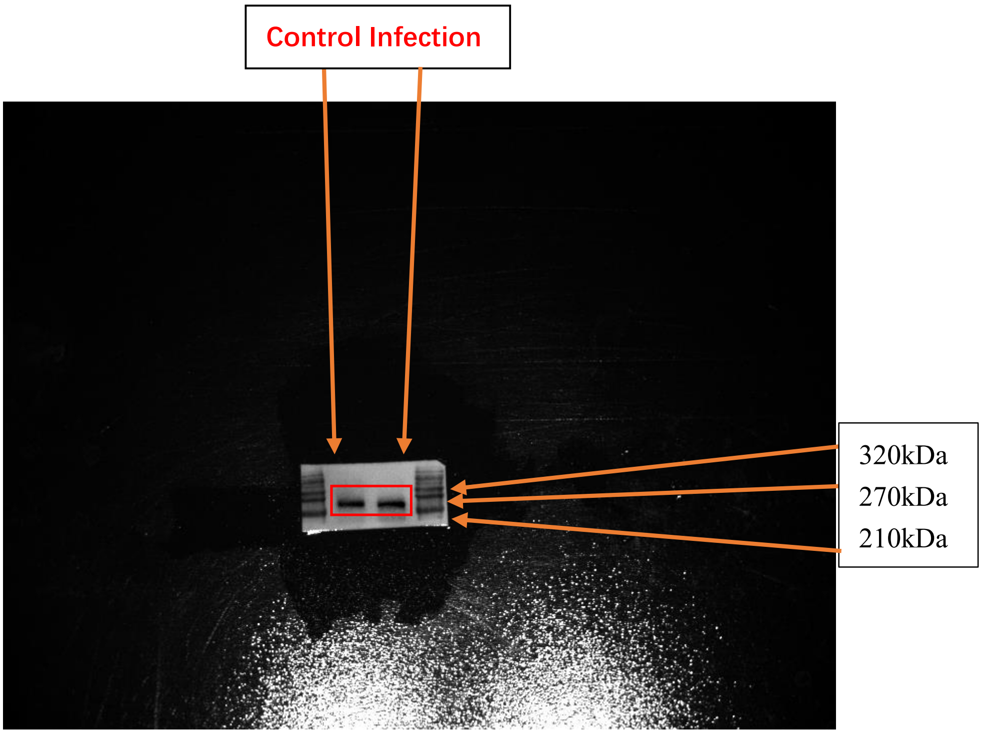
Control Infection
320kDa
270kDa
210kDa

Fig 7.D

GAPDH

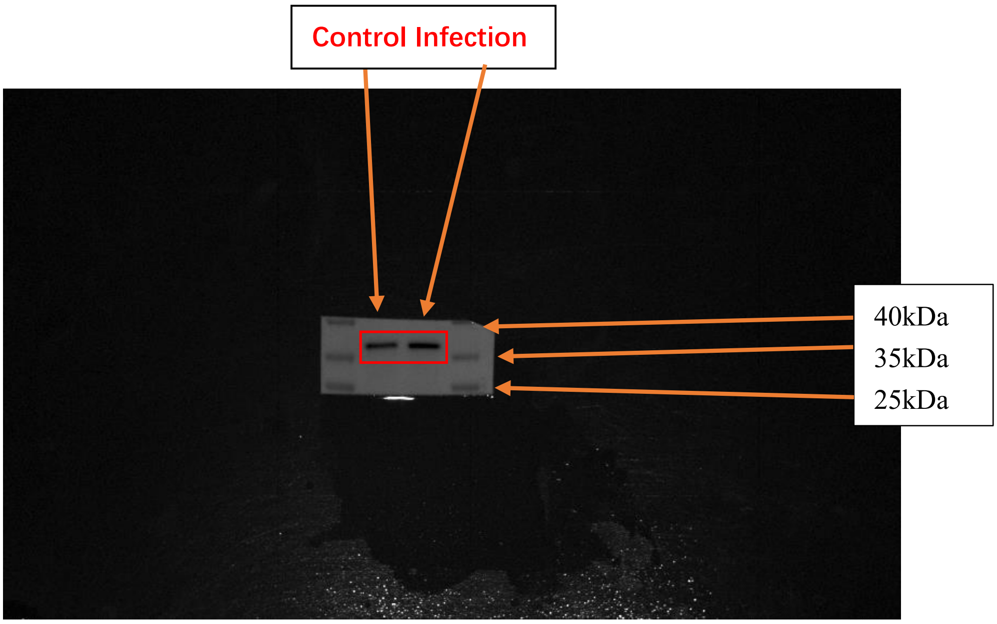
Control Infection
40kDa
35kDa
25kDa

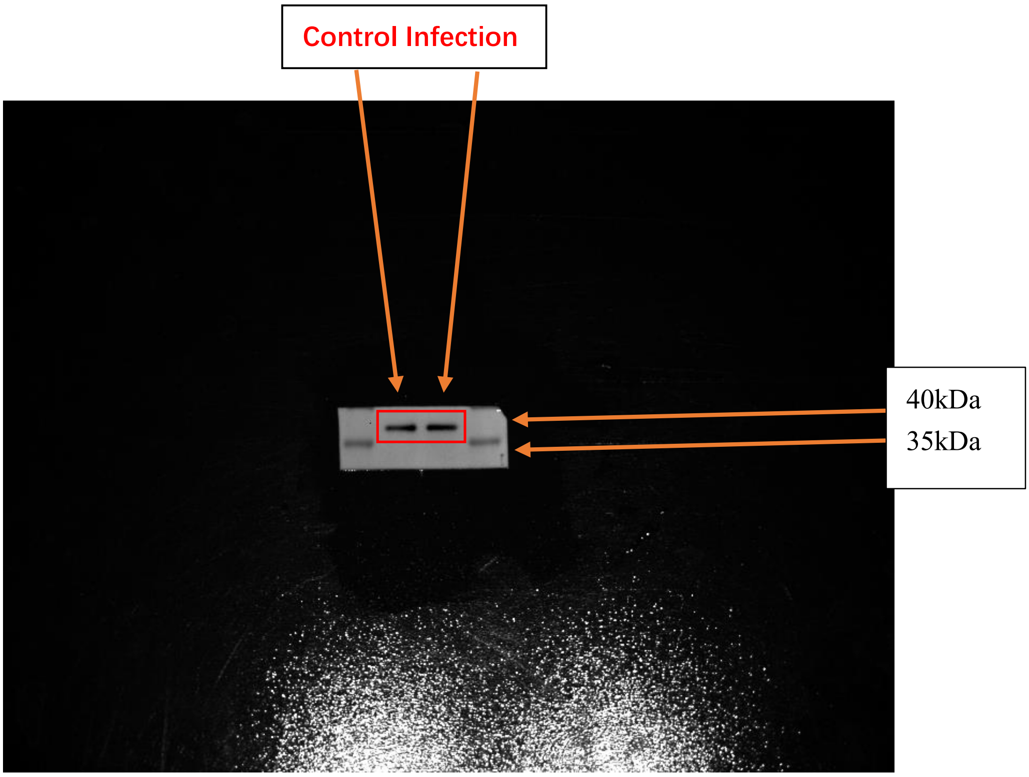
Control Infection
40kDa
35kDa

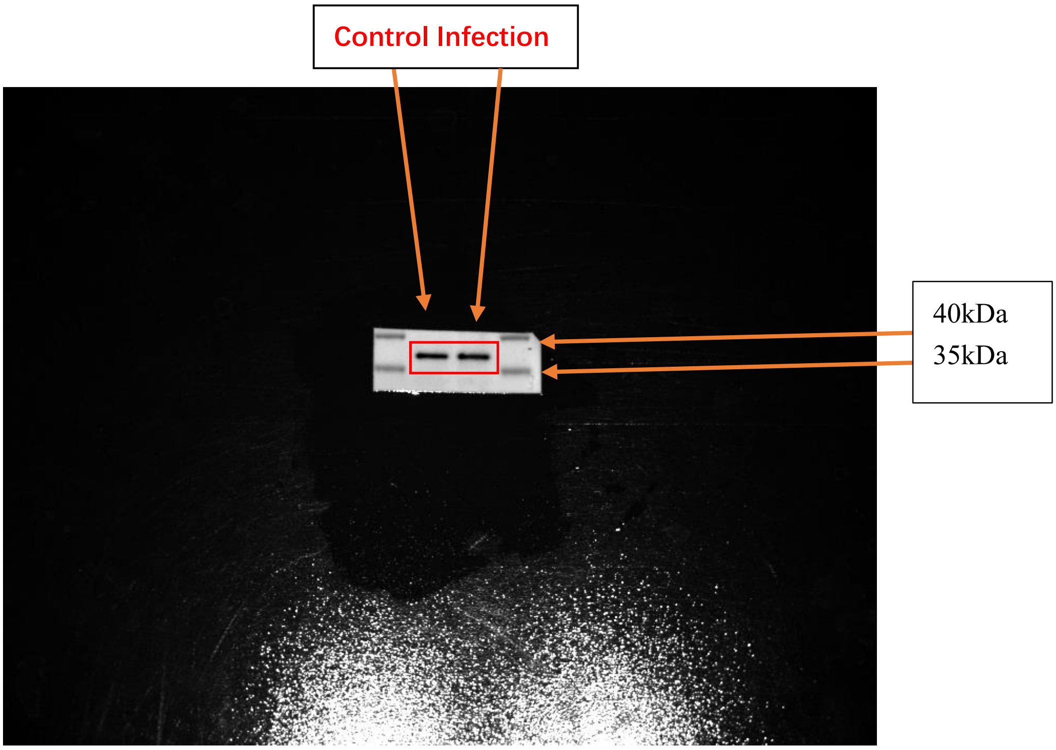
Control Infection
40kDa
35kDa
